# Data-Driven Analysis of Hole-Transporting Materials for Perovskite Solar Cells Performance

**DOI:** 10.1021/acs.jpcc.2c04725

**Published:** 2022-07-29

**Authors:** Marcos del Cueto, Charles Rawski-Furman, Juan Aragó, Enrique Ortí, Alessandro Troisi

**Affiliations:** † Department of Chemistry, University of Liverpool, Liverpool L69 3BX, U.K.; ‡ Instituto de Ciencia Molecular (ICMol), Universidad de Valencia, Catedrático José Beltrán 2, Paterna 46980, Spain

## Abstract

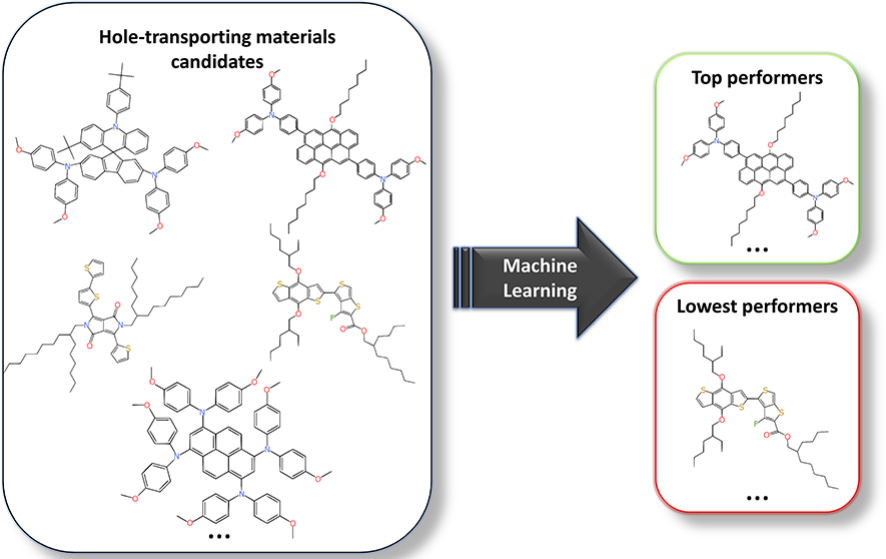

We have created a dataset of 269 perovskite solar cells, containing information about their perovskite family, cell architecture, and multiple hole-transporting materials features, including fingerprints, additives, and structural and electronic features. We propose a predictive machine learning model that is trained on these data and can be used to screen possible candidate hole-transporting materials. Our approach allows us to predict the performance of perovskite solar cells with reasonable accuracy and is able to successfully identify most of the top-performing and lowest-performing hole-transporting materials in the dataset. We discuss the effect of data biases on the distribution of perovskite families/architectures on the model’s accuracy and offer an analysis with a subset of the data to accurately study the effect of the hole-transporting material on the solar cell performance. Finally, we discuss some chemical fragments, like arylamine and aryloxy groups, which present a relatively large positive correlation with the efficiency of the cell, whereas other groups, like thiophene groups, display a negative correlation with power conversion efficiency (PCE).

## 1 Introduction

Photovoltaic cells have been gaining popularity as a renewable source of energy and, among the different types, perovskite solar cells (PSCs) have emerged as low-cost alternatives to traditional silicon-based devices. Since the seminal work by Miyasaka and co-workers in 2009,^1^ the power conversion efficiencies (PCEs) for PSC-based devices have exceptionally risen from the initial 3.8% to values over 25%.^2^ This unprecedented enhancement is due to the outstanding intrinsic features of perovskites, including wide light absorption (from visible to near-infrared), tunable bandgap energy, long electron/hole diffusion lengths, high charge-carrier mobilities, and also solution-processed fabrication.^3,4^ Nevertheless, PSCs still have stability issues. To overcome this limitation, the common organic–inorganic metal-halide perovskites can be compositionally engineered (i.e., by modifying both the cations and anions of the ABX_3_ stoichiometry) to obtain novel and enhanced perovskite materials with improved stability and efficiency.^4−6^ In the most widely used device architecture, the active perovskite is infiltrated in a mesoporous scaffold of TiO_2_ and subsequently covered by a layer of a hole-transporting material (HTM). The hole-transporting layer extracts the positive charges from the perovskite and blocks the electron movement, thus minimizing charge recombination, and also improves the stability of the perovskite layer by protecting it against moisture and oxygen.^7,8^ In this regard, the quest for novel, low-cost and efficient HTMs for their implementation in PSCs is an active research field.

In the last years, there have been significant efforts to synthesize a wide variety of HTMs with different structural motifs for PSC applications, as discussed in several recent reviews.^9−13^ Despite hundreds of HTMs being available, their impact on the efficiency of the PSCs is not straightforward. From a computational perspective, there have been different attempts^14−20^ to understand the role of HTMs in the efficiency of PSCs by analyzing the structural and electronic features of the targeted HTMs with quantum-chemical calculations. However, these studies tend to be computationally expensive and limit the number of molecules one can study at once. It is therefore difficult to extract valuable structure–property guidelines for a better rational design of novel and enhanced HTMs beyond the trial-and-error approach. A possible way of overcoming this limitation is taking advantage of the available data in the literature and training a machine learning (ML) model to detect correlations between relevant HTM properties and PCEs of the PSC-based devices. Similar approaches have been used, for example, in the context of organic solar cells^21−23^ and other energy materials.^24−28^

To date, the ML models applied in the context of PSCs^29,30^ have been mainly focused on the prediction of perovskite properties, like the band gap,^31,32^ stability,^33,34^ ionic conductivities,^35^ and other transport properties.^36^ Only a few models have tried to predict solar cell performance.^37−39^ All of these ML models focused on the perovskite, and the reduced number of HTMs limited the scope of the models to link the role of the HTM with the performance of the cell. However, in recent years, the number and types of HTMs have shown a significant increase, and we are currently able to gather hundreds of experimental PCE values from the literature. In this paper, we build an ML model to predict the PCE of the cell, using a series of features describing the properties of the HTM (fingerprints, structural properties, electronic properties, and additives), as well as the perovskite type and cell architecture. This model can be used to screen possible candidate HTMs, identifying those that are more likely to have a larger PCE, although it should be noted that in practice, one might want to consider other factors like stability or synthesizability.^40^

**Figure 1:**
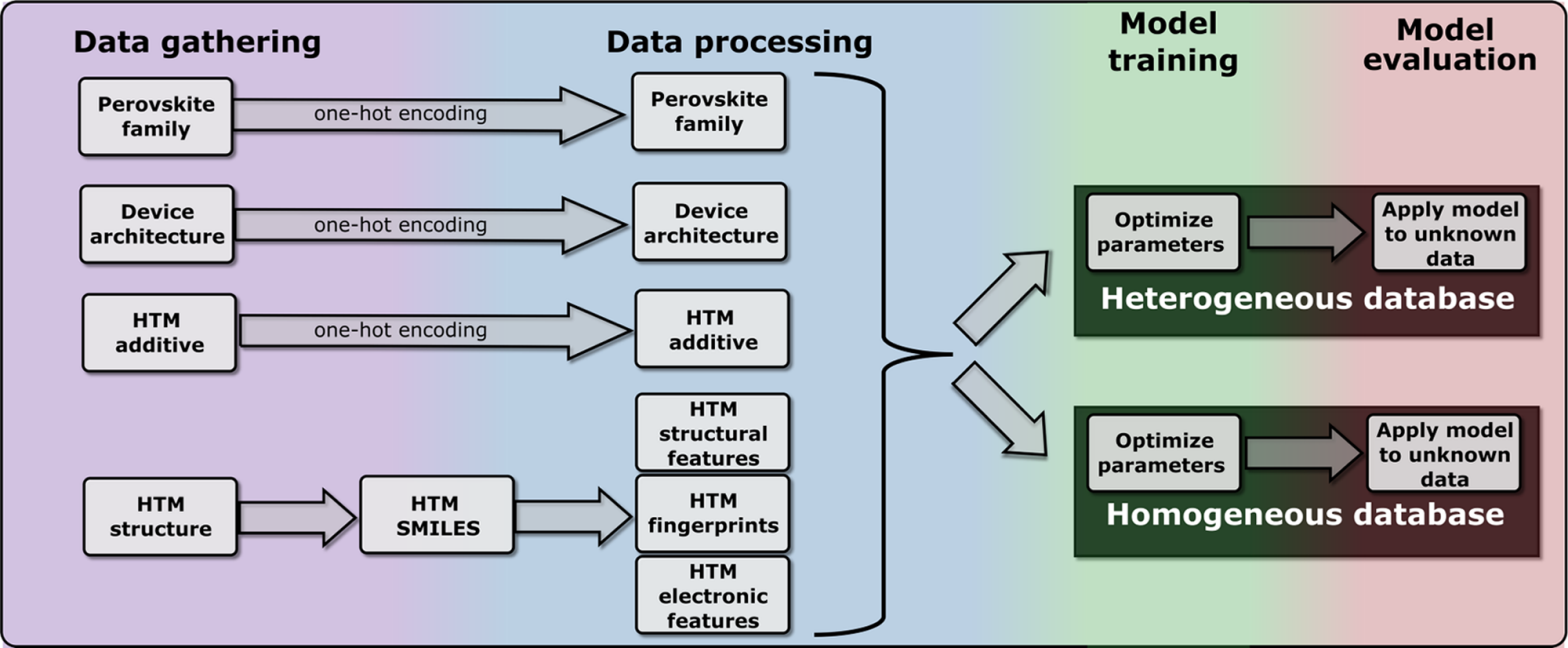
Workflow used to construct the database and train/evaluate the predictive ML model.

We first describe how we gather and process the data, paying attention to alternative ways to partition the data set. Then, we briefly describe how we built our ML model, how we trained it, and how the model was evaluated with unknown data. We finally have a discussion on the distribution of different chemical groups present in cells with large PCE and how one can use the fingerprints to analyze what chemical groups are positively and negatively correlated with cell efficiency. A schematic representation of this workflow is shown in Figure 1.

## 2 Methods

We have gathered data for a total of 269 perovskite solar cells, including the PCE, perovskite type, device architecture, HTM structures, and additives from the literature. Data are sourced from multiple review articles published in the last 6 years,^9−13^ eliminating repeated data points, data that did not provide complete HTM structure or perovskite information and outliers in the PCE < 5% region, where only a few points are available and would be challenging to make predictions. We show the chemical structure of all HTMs in Figure S7 in the Supporting Information (SI). In addition, we offer the complete database and the analysis code in a public repository.^41^

### 2.1 Perovskite Features

**Figure 2:**
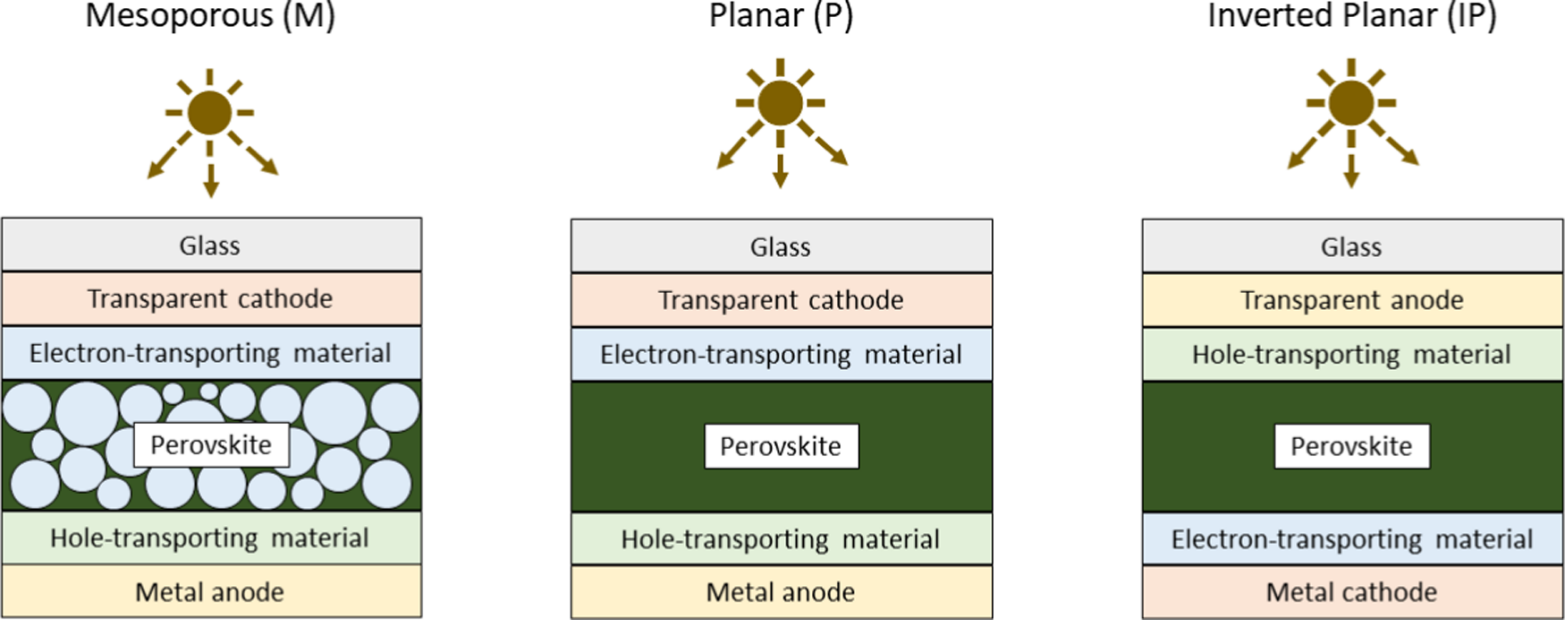
Schematic representation of the three perovskite solar cell architectures present in our database: mesoporous (M), planar (P), and inverted planar (IP).

We have classified the perovskites in our dataset in several families, broadly matching the literature classification,^42^ depending on their composition: methylammonium (MA) lead halide perovskites are labeled as family 1 (MAPbX_3_, X = Cl, Br, I), mixed-halide perovskites as family 2 (MAPbX_*x*_I_3–*x*_, X = Cl, Br), and different mixed-cation perovskites as family 3 ([FAPbI_*x*_X_3–*x*_]_1–*y*_[MAPbX_3_]_*y*_, FA *=* formamidinium, X = Cl, Br, I), family 4 ([MA_*x*_FA_1–*x*_]Pb[I_*w*_Br_3–*w*_]), family 5 (Cs_*y*_[FA_*x*_MA_1–*x*_]_1–*y*_Pb[I_*w*_Br_3–*w*_]), and family 6 (FA_*x*_Cs_1–*x*_PbI_*y*_Br_3–*y*_). To further classify the perovskites in the PSCs, we group them as a function of the device architecture:^43^ mesoporous (M), planar (P), or inverted planar (IP)—sketched in Figure 2.

**Figure 3:**
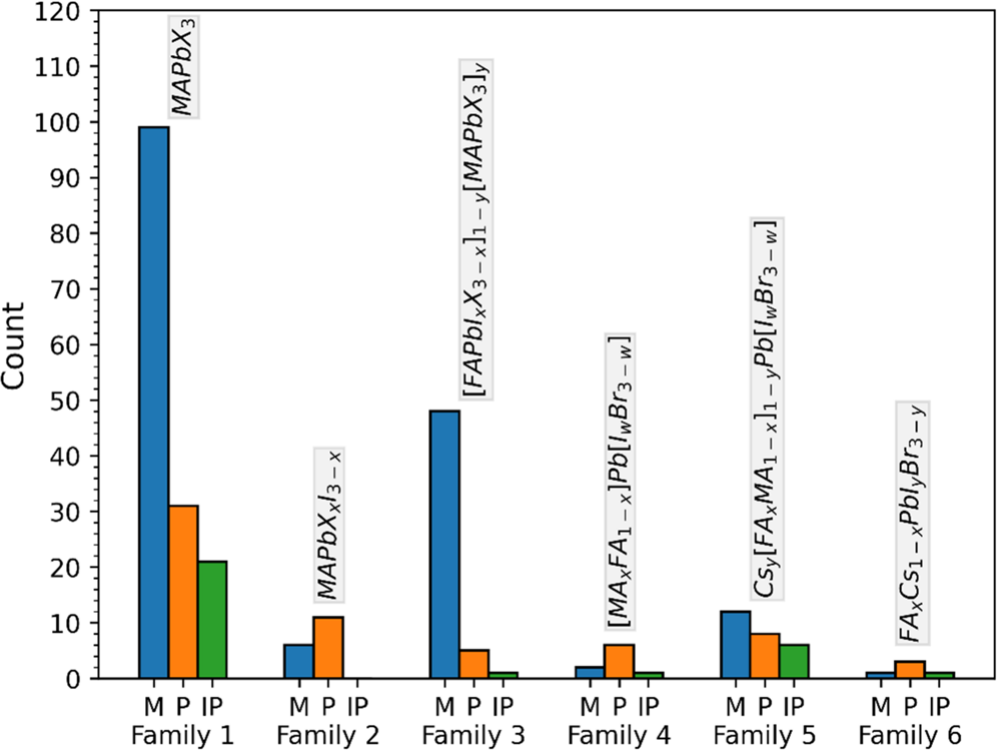
Number of data points of each perovskite family and architecture in our database.

We show the number of data points in our database belonging to each family and architecture in Figure 3, where one can observe how the database presents a heterogeneous distribution of perovskite family/architecture. Our model accounts for this heterogeneity, and each PSC has a one-hot encoded^44^ vector of length 6 indicating its perovskite family and a one-hot encoded vector of length 3 indicating its device architecture. However, to isolate the effect of the HTM on the PSC efficiency, we have considered a subset of the total database, in which we only consider the most numerous class involving the same perovskite family and device architecture. Thus, we are considering two different databases:

- Heterogeneous database. A total of 269 PSCs, with six different families of perovskites and three possible architectures.
- Homogeneous database. A total of 100 PSCs, with the standard MAPbX_3_ perovskite and a mesoporous architecture.

### 2.2 HTM Features

Each HTM molecular structure is encoded into a SMILES string (a simplified molecular-input line-entry system that defines the structure of a chemical species as a short text string).^45^ The SMILES of each HTM is used to calculate different HTM features:

- Molecular fingerprints: The chemical structure of the HTMs is expected to influence the efficiency of the solar cell, which motivates us to use their Morgan fingerprints.^46^ These fingerprints are bit-vectors indicating the presence or absence of specific fragments within the HTM, and thus can be used to describe the chemical similarity between our HTMs. We have used a radius of 2 and a length of 2048 bits per fingerprint.
- Structural features: We explicitly included additional structural features that might affect the efficiency of the cell. We show in Section S1 in the SI the list of the 32 structural features we initially considered, which include the number of specific chemical groups (e.g., porphyrin, triphenylamine (TPA), carbazole, etc.) and other structural properties obtainable from the HTM SMILES (e.g., number of rotatable bonds, number of stereocenters, number of heteroatoms, etc.). As shown in the SI, some of these features are strongly correlated, and we reduced the number of relevant structural features to 24 by eliminating those with a high correlation (*r* > 0.7). After performing a recursive feature elimination, we obtain the best performance when using 12 structural features with the heterogeneous database (number of phthalocyanine groups, number of porphyrin groups, number of carbazole groups, number of triphenylamine groups, whether HTM is a polymer or not, number of acenaphthene groups, number of benzotrithiophene groups, number of silicon atoms, number of rotatable bonds, number of aliphatic carbocycles, number of aliphatic heterocycles and number of stereocenters), and using nine structural features with the homogeneous database (number of spiro atoms, number of carbazole groups, number of triphenylamine groups, number of acenaphthene groups, number of benzotrithiophene groups, number of sp^3^ carbon atoms, number of aliphatic heterocycles, number of aromatic heterocycles and molecular planarity—calculated as the sum of atomic distances from the plane of best fit).^47^
- Electronic features: To account for possible limitations of the fingerprints and structural features, which are based just on the molecular topology and connectivity, we have included features obtained from the HTM electronic structure. Initially, we considered four electronic features: (i) highest occupied molecular orbital (HOMO) energy (*E*_HOMO_), which is expected to be correlated to the PCE; (ii) lowest unoccupied molecular orbital (LUMO) energy (*E*_LUMO_), which might be relevant since the HTM *E*_LUMO_ should be higher than the conduction band of the perovskite to act as an electron blocker; (iii) ionization potential (IP), which might affect the generation of a hole; and (iv) reorganization energy for oxidation (λ), which largely influences the charge transport behavior of the HTM. As shown in Figure S5 in the SI, the IP and *E*_HOMO_ are (as expected) strongly correlated, so we finally only considered *E*_HOMO_, *E*_LUMO_, and λ. Starting from each HTM SMILES, we optimized the most stable conformer at the semiempirical PM7^48^ level, as described in Section S2 in the SI, and we used that geometry as a starting point to optimize the geometry of the neutral molecule and cation at the B3LYP/3-21G* level, which has been shown to reproduce similar trends to larger basis sets.^49^ We then performed single-point calculations of the neutral molecule and cation using CH_2_Cl_2_ as a solvent within the polarized continuum model (PCM) at the B3LYP/6-31G** level, with the Gaussian16 software.^50^
- HTM additives: It is common to dope the HTMs with different additives to increase their hole mobility. We have considered the presence or absence of 10 different additives for each HTM in our database: Li-TFSi, t-BP, FK102, FK209, FK269, F4-TCNQ, MY11, Et_4_N-TFSi, H-TFSi, and 2-Py. These additives have been one-hot encoded, so each data point in the database has a binary array of length 10 describing its HTM additives.

### 2.3 Machine Learning model

We have used Kernel Ridge Regression (KRR), as implemented in Scikit-learn,^51^ to build our predictive model. The KRR algorithm has been previously used to predict other properties of PSCs^52,53^ and organic solar cells,^54,55^ being particularly effective for multidimensional datasets with a limited amount of data. The algorithm combines ridge regression with the kernel trick and is able to approximate nonlinear functions. Given a training set defined by {***x***_*i*_} features and ***y*** target property values (PCE in our case), the target property of a new data point (*y*′) is approximated as
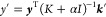
1where α is the regularization parameter, *I* is the identity matrix, *K* is the kernel matrix defined as *K*_*i,j*_*= f*(***x***_*i*_*,**x***_*j*_), and *k*′_*i*_*= f*(***x***_*i*_*, **x***′). The function *f* is the kernel function, which effectively measures the similarity between different data points in the feature space. Our kernel function is defined as:
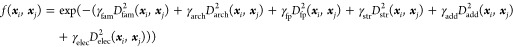
2where the γ_*i*_ parameters indicate the weight of each distance, and the *D*_*i*_(***x***_*i*_*,**x***_*j*_) terms measure the distance of the different features:

- *D*_fam_: measured as the Euclidean distance between the one-hot-encoded vectors indicating the perovskite family of each PSC.
- *D*_arch_: measured as the Euclidean distance between the one-hot-encoded vectors indicating the device architecture of each PSC.
- *D*_fp_: measured from the Tanimoto similarity index (*T*)^56^ between two fingerprint vectors as *D*_fp_(***x***_*i*_,***x***_*j*_) = 1 – *T*(***x***_*i*_^fp^,***x***_*j*_^fp^).
- *D*_str_: measured as the Euclidean distance between the vectors containing all structural features (after standardizing them to have a mean value of 0 and a standard deviation of 1).
- *D*_add_: measured as the Euclidean distance between the one-hot-encoded vectors indicating the presence/absence of each HTM additive.
- *D*_elec_: measured as the Euclidean distance between the vectors containing all electronic features (after standardizing them to have a mean value of 0 and a standard deviation of 1).

Note that having these different features with different weights in the kernel allows us to see their contribution to the final model, as well as control which features are considered in the model since they represent unique properties and they might have different availability. In our model, we have used 90% of our data as a training set and used the other randomly chosen 10% of our dataset as a test set. We have used a leave-one-out cross-validation (LOO-CV) to optimize the γ_*i*_ parameters and the regularization parameter α, by minimizing the root-mean-square error (rmse) of our prediction in the training set. Finally, the optimized parameters are used to evaluate the PCE of the data points in the test set. The parameters of the model are optimized using a differential evolution algorithm.^57^ The details of this procedure, along with the optimized parameters, are shown in Section S3 in the SI.

## 3 Results and Discussion

**Figure 4:**
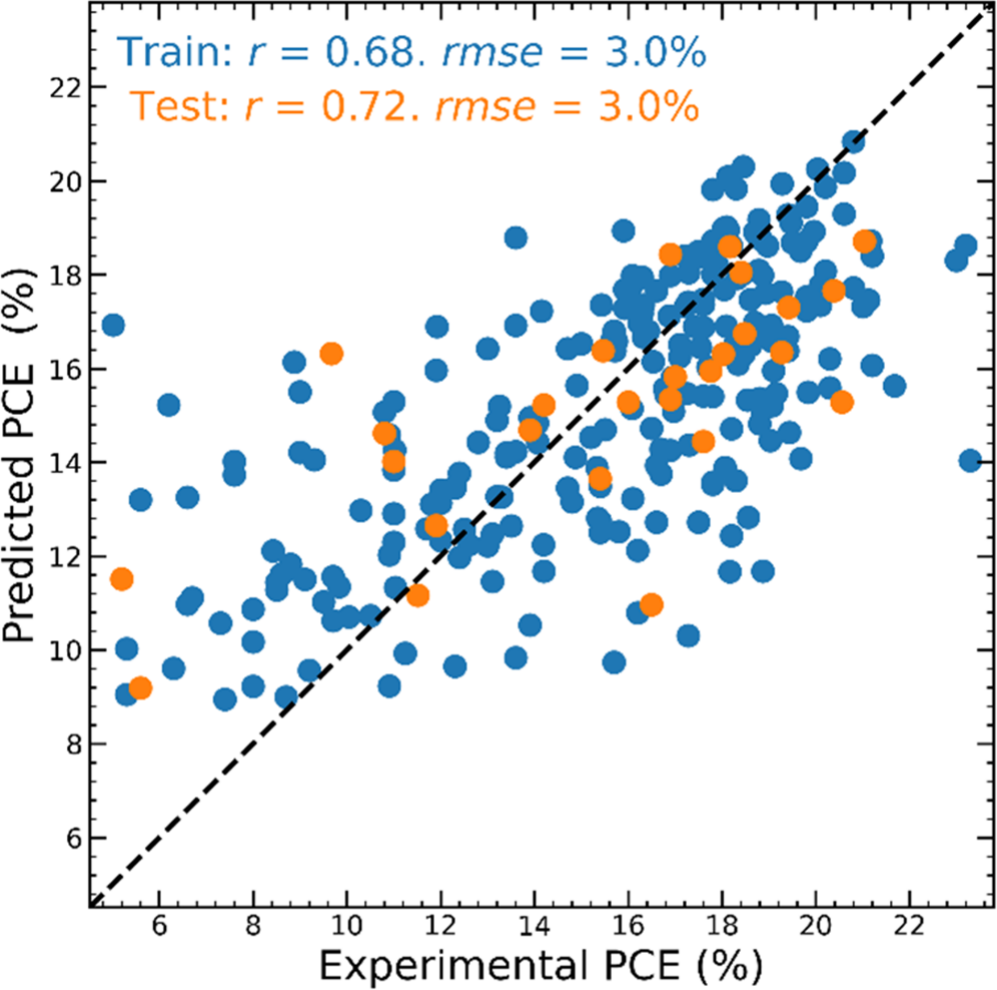
Experimental and predicted PCE of data in the heterogeneous database. Blue points correspond to data in the training set, and orange points correspond to data in the test set.

We first discuss our ML model with the heterogeneous database. In Figure 4, we show the predicted PCE and experimental PCE values for the 242 points in the training set, as well as the 27 points in the test set. Both sets result in a very similar rmse = 3.0% and Pearson correlation (*r*_train_ = 0.68 and *r*_test_ = 0.72). These results are very promising and suggest that one could use this model to screen unknown HTM candidates. However, the errors for some of these points are relatively large (>5%), so we also discuss how one can use this ML model to make qualitative predictions that might accelerate the screening of novel HTM candidates. For example, we can split our test set into two groups: top-performance HTMs (14 points with the highest PCE) and lowest-performance HTMs (13 points with the lowest PCE). Then, we can rank our predictions and see if top-performance HTMs are correctly predicted in the experimental top-14. We show both the experimental and predicted PCE values in Table 1, where the HTM number (HTM no.) corresponds to the list shown in Figure S7. We can observe how our model correctly predicts 12 out of the top-14 HTMs as top-performers and 11 out of the lowest-13 HTMs as lowest-performers.

**Table 1:** Experimental and Predicted PCE Values for Each of the HTMs in the Test Set of the Heterogeneous Database^a^

HTM no.	experimental PCE	predicted PCE	experimental rank order	predicted rank order
9	21.04	18.72	1	1
15	20.56	15.29	2	15*
16	20.38	17.66	3	5
36	19.42	17.31	4	6
42	19.27	16.35	5	9
67	18.48	16.75	6	7
72	18.40	18.05	7	4
81	18.17	18.60	8	2
91	18.03	16.32	9	10
100	17.76	15.94	10	12
106	17.60	14.44	11	20*
121	17.00	15.83	12	13
125	16.90	18.44	13	3
126	16.90	15.35	14	14
137	16.50	10.96	15	26
151	16.00	15.28	16	16
162	15.46	16.38	17	8*
166	15.40	13.66	18	22
177	14.20	15.23	19	17
185	13.90	14.68	20	18
213	11.90	12.65	21	23
217	11.51	11.17	22	25
224	11.00	14.02	23	21
229	10.80	14.61	24	19
237	9.67	16.32	25	11*
265	5.60	9.19	26	27
268	5.20	11.51	27	24

a Ranked order for experimental and predicted values corresponds to the sorted values in decreasing order. Values marked with * are incorrectly categorized as top- or lowest-performer.

**Figure 5:**
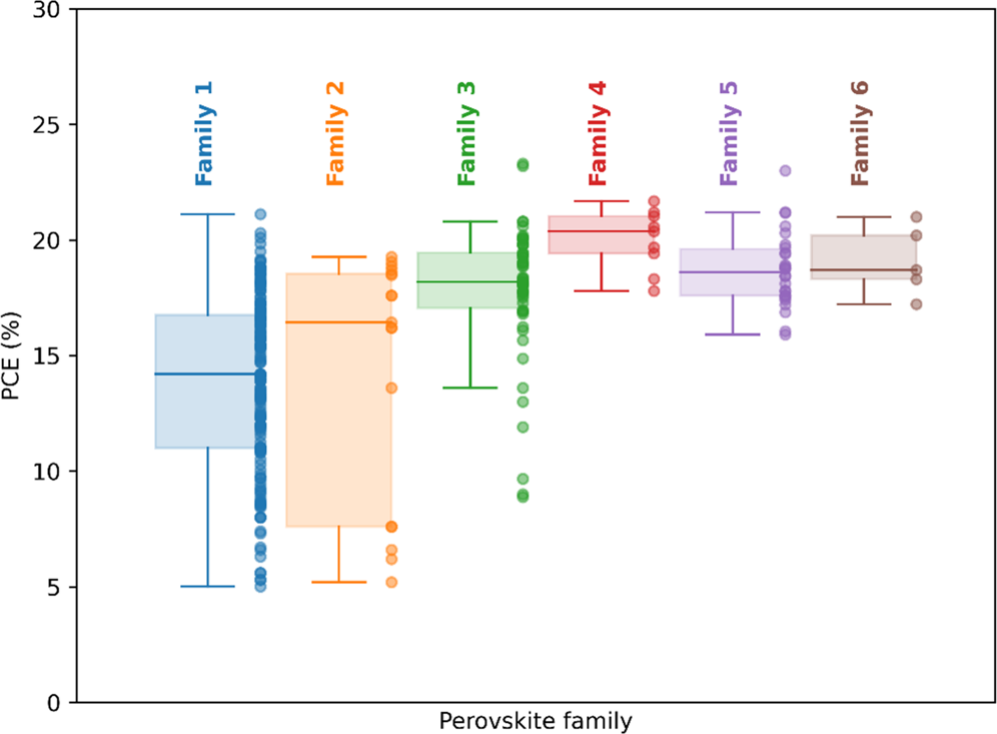
PCE distribution in our heterogeneous database as a function of the perovskite family.

However, it should be noted that the perovskite families 2–6 only have a few data points, as shown in Figure 3. In addition, families 2–6 tend to have a narrow PCE distribution, as shown in Figure 5, which means that simply predicting the average performance of these families would return accurate results, even when ignoring the effect of the HTM. These data clusters are not uncommon in experimental datasets since negative results are rarely reported and datasets tend to have clusters of high-performing points, which affects the performance of the predictive model.^58^ There have been attempts to mitigate these biases, like including data from failed experiments,^59^ optimizing the design of experiments,^60^ or adopting novel frameworks.^61,62^ In our case, we can reduce the complexity of the problem and isolate the effect of the HTM simply by working with our homogeneous dataset, which corresponds to a data subset with the most numerous class—family 1 (MAPbX_3_) and mesoporous architecture. Note that if more data becomes available for some of the less populated families and architectures, one could also try to improve the performance by doing a more sophisticated encoding of the perovskite layer and considering additional features with its composition and properties.

**Figure 6:**
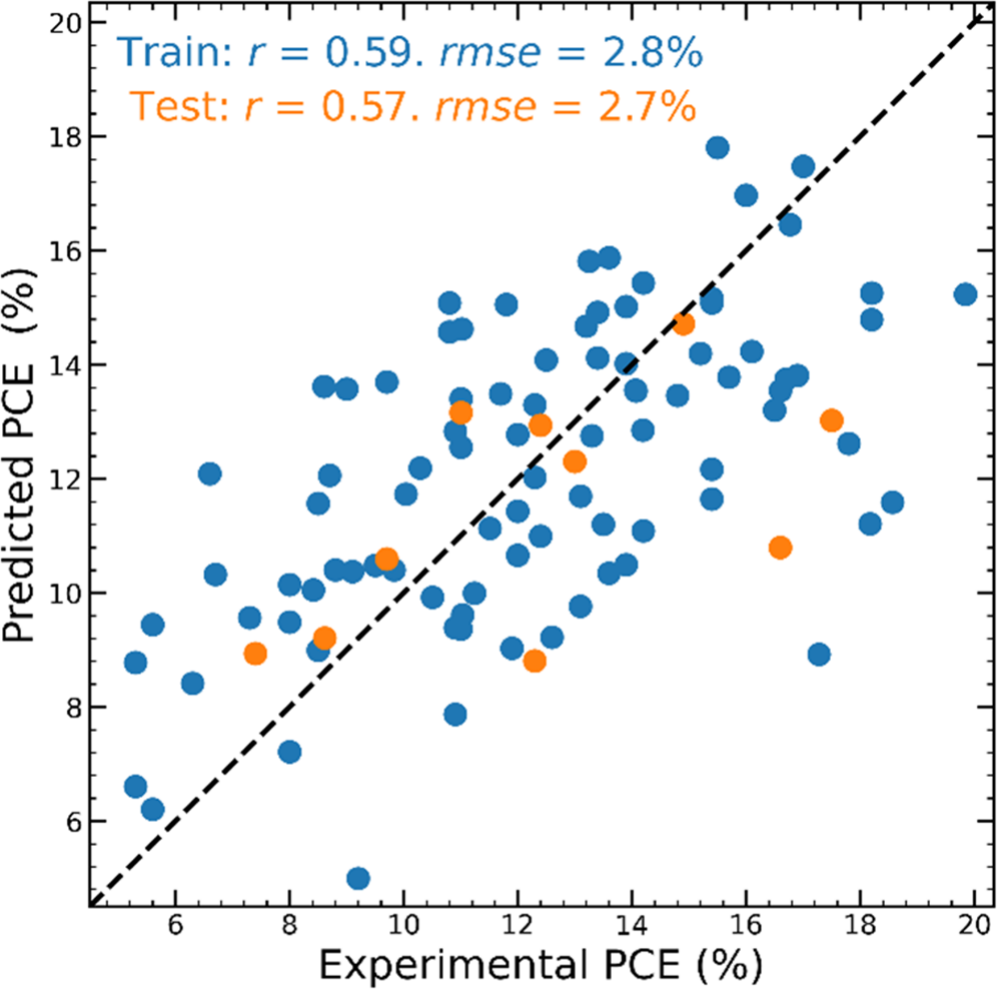
Experimental and predicted PCE of data in the homogeneous database. Blue points correspond to data in the training set, and orange points correspond to data in the test set.

When working with the homogeneous database, we reoptimize the model parameters to minimize the rmse in the training set. As in the case of the heterogeneous database, both training and test sets produce very similar results (rmse_train_ = 2.8% and rmse_test_ = 2.7%) and Pearson correlation (*r*_train_ = 0.59 and *r*_test_ = 0.57), as shown in Figure 6.

**Figure 7:**
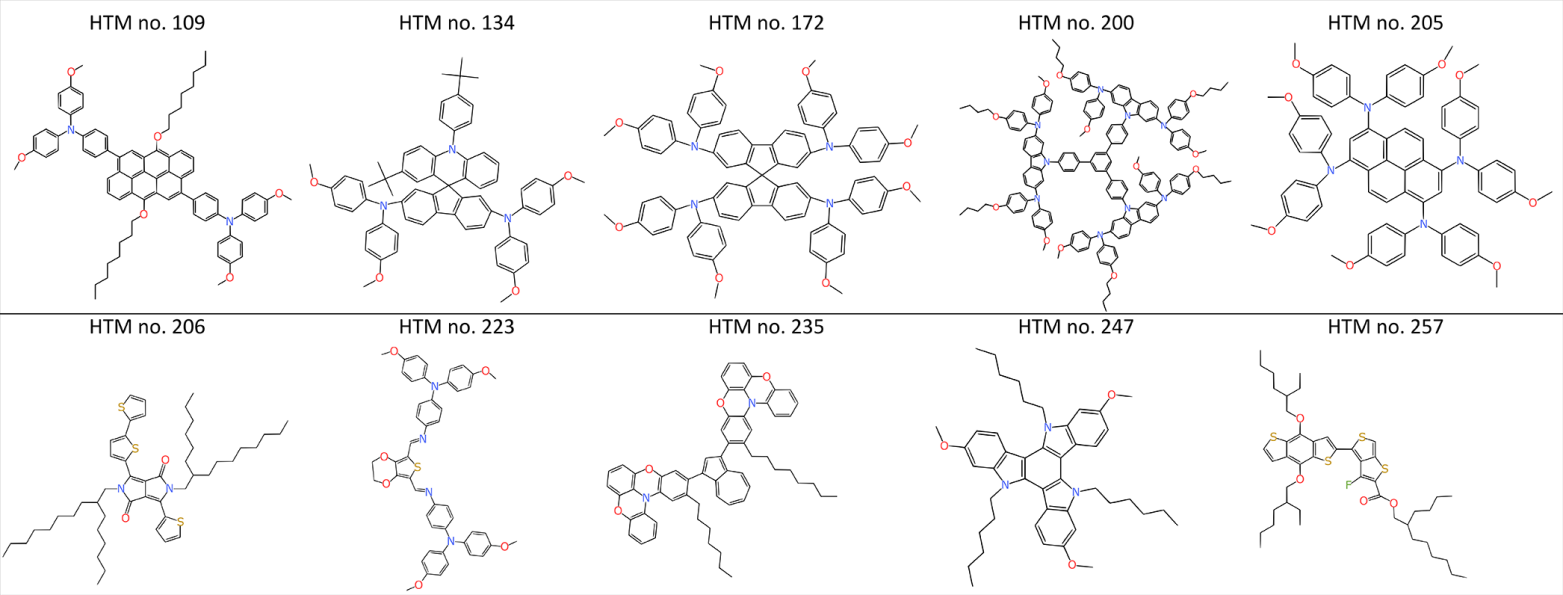
Chemical structures of the 10 HTMs used as a test set to evaluate our ML model with the homogeneous database.

Although the prediction error committed in some of these HTMs is not negligible, the model still returns a moderate correlation when predicting the efficiency of unknown cells (*r* = 0.57). We show in Table 2 the experimental and predicted PCE values of the 10 HTMs in the test set of the homogeneous database. As we did with the hetereogeneous database, we can split our test set as top-performers and lowest-performers (top-5 HTMs and lowest-5 HTMs, respectively). Then, if we rank our predicted PCE values, we can observe how four out of five of our top-5 predictions are within the experimental top-5. Similarly, four out of five of the lowest-5 predictions are within the experimental lowest-5. The chemical structures for these 10 HTMs are shown in Figure 7.

**Table 2:** Experimental and Predicted PCE Values for Each of the HTMs in the Test Set of Our Homogeneous Database^a^

HTM no.	experimental PCE	predicted PCE	experimental rank order	predicted rank order*
109	17.50	13.03	1	3
134	16.60	10.80	2	6*
172	14.90	14.72	3	1
200	13.00	12.30	4	5
205	12.40	12.94	5	4
206	12.30	8.81	6	10
223	11.00	13.16	7	2*
235	9.70	10.60	8	7
247	8.62	9.21	9	8
257	7.40	8.94	10	9

a Ranked order for experimental and predicted values corresponds to their respective PCE values sorted in decreasing order. Values marked with * are incorrectly categorized as top- or lowest-performer.

Finally, it is worth noting that, because our predictive model is formed by several types of features (e.g., fingerprints, structural and electronic features), we can explore how our predictions are affected when using only one of these features at a time (along with the HTM additives, to account for data points that might have the same HTM, but a different additive, affecting the PCE). We show these cases in Table 3, along with the prediction results when combining all features. We observe how using only structural or electronic features results in large rmse and low *r*, which indicates that they are not suitable to predict PCE by themselves. However, when we use only fingerprints, the rmse and *r* improve significantly, which tells us that this simple feature can account for most of the correlation in our data, and one could build a reasonably accurate model with just the fingerprints, which has also been observed in other organic photovoltaic materials.^54^ Combining all features results in a marginal increase of the model performance, although it comes at the cost of adding more computationally expensive features. The optimized parameters and predicted values are shown in Section S3 in the SI.

**Table 3:** Values of rmse and *r* When Training the Model with Different Sets of Features with the Homogeneous Database^a^

features	rmse (%)	*r*
fingerprint + additives	2.8 (2.8)	0.59 (0.54)
structural + additives	6.9 (3.9)	0.19 (0.27)
electronic + additives	4.1 (5.4)	0.40 (0.65)
fingerprint + structural + electronic + additives	2.8 (2.7)	0.59 (0.57)

a Values outside the parentheses correspond to the training set, and those within parentheses correspond to the test set.

Since most of our model performance is due to the information encoded in the fingerprint, we performed an analysis to detect if any fragment is significantly correlated with PCE. For each of the 2048 bits in the fingerprints, we studied the correlation between its presence in each molecule of the dataset and the corresponding PCE value with a point biserial correlation coefficient, *r*, which is equivalent to Pearson’s correlation when one variable is binary.

**Figure 8:**
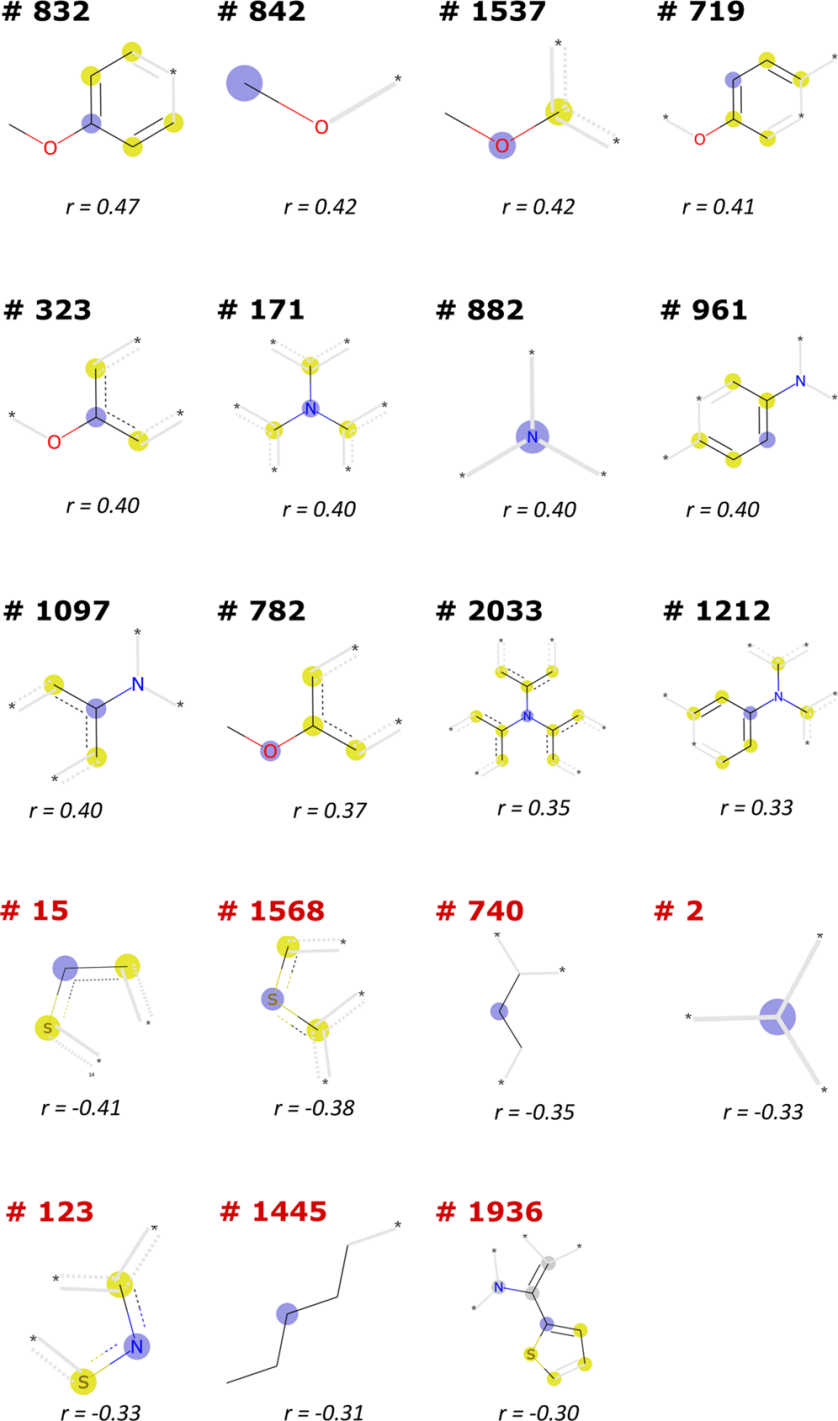
Fragments corresponding to the bits of the fingerprint which have a correlation coefficient *r* > 0.30 (top) and *r* < −0.30 (bottom). Bits representations were obtained with the RDKit package.^64^

When we focus on the homogeneous database, we observe that there are 19 bits with |*r*| > 0.30, which are shown in Figure 8. In Section S4 in the SI, we show the *p*-value corresponding to this measurement (below 0.003 in all cases) and the 95% confidence interval for *r*, showing that there is statistical significance to these trends. We also show in the SI the results of a Mann–Whitney *U* test for each of these fragments, which measures the statistical difference between the PCE values of the molecules without the fragment and the PCE values of the molecules with the fragment, confirming that there is a statistically significant correlation between the presence of these fragments and the PCE values.

We can observe in Figure 8 how most of the bits with a positive correlation correspond to conjugated fragments with oxygen and nitrogen (e.g., arylamine and aryloxy groups) that form part of the triphenylamine (TPA) or diphenylamine (DPA) peripheral units present in many HTMs. This statistical analysis performed over many HTMs therefore supports the empirical trend that arylamine units with alkoxy chains are important for efficient HTMs for PSC devices. On the other hand, it is worth noting that the fragments with a negative correlation with PCE are alkane chains (bit #740, #2, and #1445) or conjugated fragments with sulfur (bit #145, #1568, #123, and #1936) like thiophene rings. This is an interesting trend since two out of five molecules with the highest PCE in the homogeneous database contain thiophene rings, the average PCE of molecules with thiophene rings in the homogeneous database is 11.7%, which is lower than the overall average PCE for this database (12.3%). This means that even if there is a significant percentage of top-performers with thiophene rings, only a small portion of molecules with this fragment are top-performers, and we observe a slight negative correlation between this group and the cell’s performance. Importantly, this negative correlation contrasts with the general conception, extracted from only a few HTM systems, that sulfur-containing conjugated systems are good HTM candidates due to favorable sulfur–perovskite interactions.^63^

The results for the heterogeneous database are very similar, and 13 of the 20 most correlated bits are the same observed in the homogeneous database. In this case, only one fragment (#1097) has a value of |*r*| > 0.3, although there are a total of 27 fragments with a correlation of |*r*| > 0.2, as shown in the SI.

## 4 Conclusions

We have gathered data of 269 perovskite solar cells, including their PCE, perovskite family, device architecture, and multiple HTM features from the literature. Constructing PSCs, or even performing accurate electronic structure calculations of the HTMs, might be very resource- and time-consuming. Therefore, we have used our database to train a predictive ML model with reasonably good accuracy, which suggests that this model would be a useful HTM screening tool. Moreover, we have shown how this model can also be used to correctly identify most of the top-performing and lowest-performing HTMs. We have discussed how the performance of the predictive model is overestimated by data clusters for different perovskite and architectures, although one can still gain insight from predictions made for HTMs corresponding to a heterogeneous distribution of perovskite and architecture. Finally, we have shown the correlation between specific molecular fragments, like arylamine and aryloxy groups, which have a large positive correlation with PCE, or thiophene groups, which have a large negative correlation with PCE. In essence, this approach enables researchers to discriminate between apparent correlations emerging for very limited observations and statistically meaningful correlations consistent with the entire dataset. We expect this type of analysis to become increasingly more powerful as more data are added to the provided dataset and more sophisticated descriptors are used, helping to increase the prediction generalizability and reduce bias effects.

## Supporting Information Available

List of features used in the model, additional details about the conformer search and ML model, *k*-nearest neighbor results, extra information on the correlation between chemical fragments and PCE, and chemical structures in the database (PDF)
