# Supplementary material for: Data-Driven Analysis of Hole-Transporting Materials for Perovskite Solar Cells Performance
Source: J Phys Chem C Nanomater Interfaces. 2022 Jul 29;126(31):13053–61. doi: 10.1021/acs.jpcc.2c04725 (PMC9376947; doi:10.1021/acs.jpcc.2c04725)
Supplement: Supplementary file 1 — jp2c04725_si_001.pdf [file jp2c04725_si_001.pdf]

Electronic supplementary information

Data-Driven Analysis of Hole-Transporting Materials for Perovskite Solar Cells Performance

Marcos del Cueto*[1], Charles Rawski-Furman[1], Juan Aragó[2], Enrique Ortí[2], Alessandro Troisi[1]

1. Department of Chemistry, University of Liverpool, Liverpool, L69 3BX, UK

2. Instituto de Ciencia Molecular (ICMol), Universidad de Valencia, Catedrático José Beltrán 2, Paterna 46980, Spain

Contents:

S1. HTM features
   a. Structural features
   b. Electronic features
S2. Conformer search
S3. ML model
   a. Differential evolution algorithm
   b. Heterogeneous database parameters
   c. Homogeneous database parameters
S4. kNN results
S5. Chemical fragments correlation with PCE
S6. Database
S7. References

S1. HTM features

a. Structural features

We initially consider the following 32 structural features:

1. Phthalocyanine
2. Porphyrin
3. Azulene
4. Spiro atoms
5. Thiophene
6. Furan
7. Fluorene
8. Carbazole
9. Dibenzofuran
10. Diphenylamine
11. Triphenylamine
12. Polymer *(boolean)*
13. Xanthene
14. Acenaphthene
15. Benzotrithiophene
16. Silicon atoms
17. Rotatable bonds
18. Molecular weight
19. sp^3 Carbons
20. Aliphatic carbocycles
21. Aliphatic heterocycles
22. Aliphatic rings
23. Amide bonds
24. Aromatic carbocycles
25. Aromatic heterocycles
26. Aromatic rings
27. Stereocenters
28. Bridgehead atoms
29. Heterocycles
30. Heteroatoms
31. Rings
32. Molecular planarity *(measured as the sum of atomic distances from plane of best fit - PBF)*[1]

The correlation matrix of all these features are shown in Figure S1 (for the heterogeneous database), and we then remove those features with a Pearson correlation larger than 0.7:

- molecular weight (large correlation with rotatable bonds)
- fluorenes (large correlation with aliphatic carbocycles)
- aliphatic rings (large correlation with aliphatic heterocycles)
- thiophenes (large correlation with aromatic heterocycles)
- aromatic rings (large correlation with aromatic carbocycles)
- rings (large correlation with aromatic carbocycles)
- heterocycles (large correlation with aromatic heterocycles)
- bridgehead atoms (large correlation with phthalocyanines)

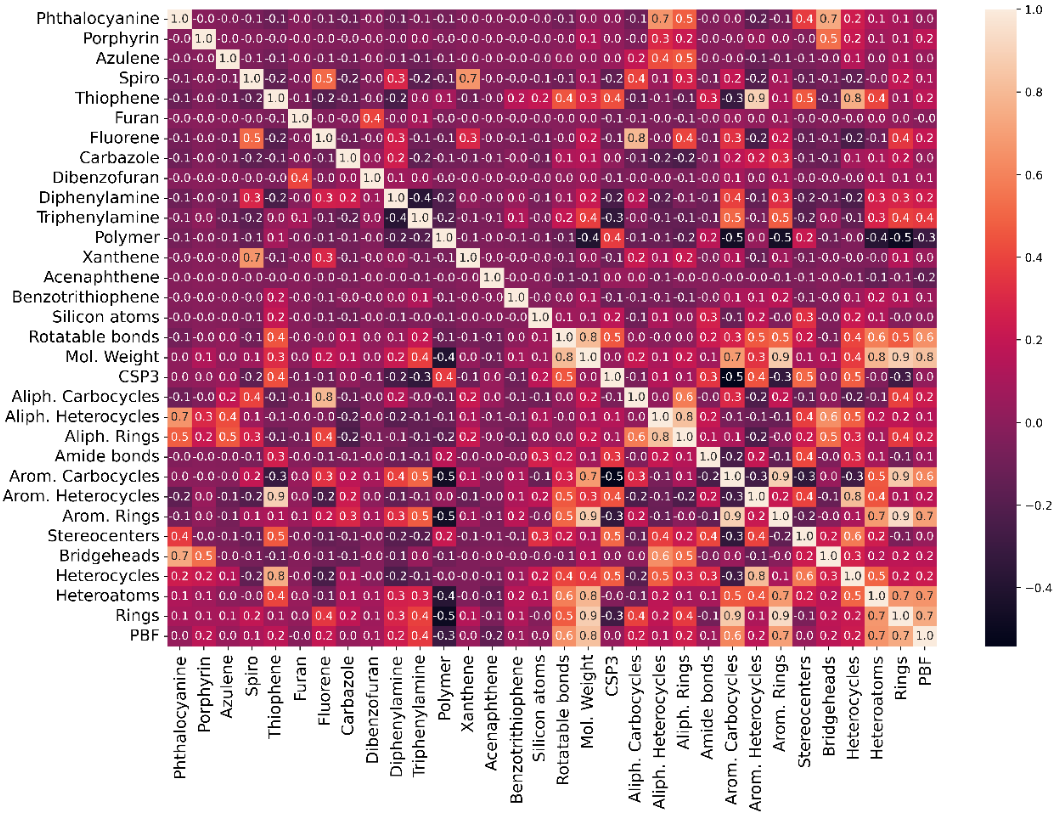

Figure S1. Correlation matrix with the initial 32 structural features in the heterogeneous dataset.

Finally, we used a *k*-nearest neighbors model, using all the 269 data points to estimate the root mean square error (rmse) when using a 10-fold cross-validation. We performed a recursive feature selection, whose results are shown in Figure S2, and the following descriptors are dropped sequentially: spiro atoms, amide bonds, azulene, diphenylamine, xanthene, aromatic carbocycles, heteroatoms, aromatic heterocycles, molecular planarity, sp^3 carbons, furan, dibenzofuran, porphyrin, aliphatic carbocycle, stereocenters, silicon atoms, phthalocyanine, benzotrithiophene, triphenylamine, acenaphthene, aliphatic heterocycles, rotatable bonds, carbazole. The lowest rmse is achieved when using 12 structural features, corresponding to:

1. Phthalocyanine
2. Porphyrin
3. Carbazole
4. Triphenylamine
5. Polymer
6. Acenaphthene
7. Benzotrithiophene
8. Silicon atoms
9. Rotatable bonds
10. Aliphatic carbocycles
11. Aliphatic heterocycles
12. Stereocenters

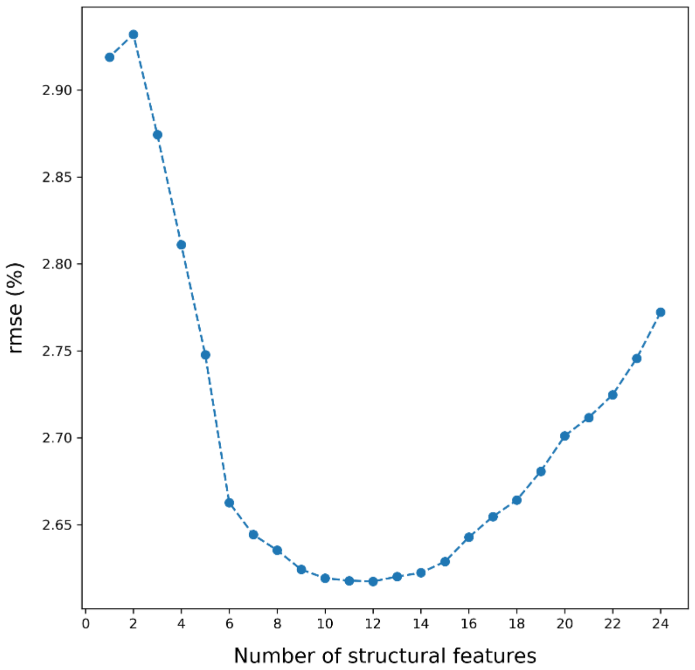

Figure S2. rmse values obtained with a feature recursive elimination in the heterogeneous database.

In the case of the homogeneous database, there is only one molecule with a phthalocyanine group, and there are no porphyrins present, so we do not consider these two features. In Figure S3, we show the resulting correlation matrix with the remaining 30 structural features. We remove the following nine features with a Pearson correlation larger than 0.7:

- Dibenzofuran (large correlation with furan)
- Molecular weight (large correlation with rotatable bonds)
- Fluorene (large correlation with aliphatic carbocycles)
- Azulene (large correlation with aliphatic heterocycles)
- Aliphatic rings (large correlation with aliphatic carbocycles and aliphatic heterocycles)
- Thiophene (large correlation with heterocycles and aromatic heterocycles)
- Aromatic rings (large correlation with aromatic carbocycles)
- Heterocycles (large correlation with aromatic heterocycles)
- Rings (large correlation with aromatic carbocycles)

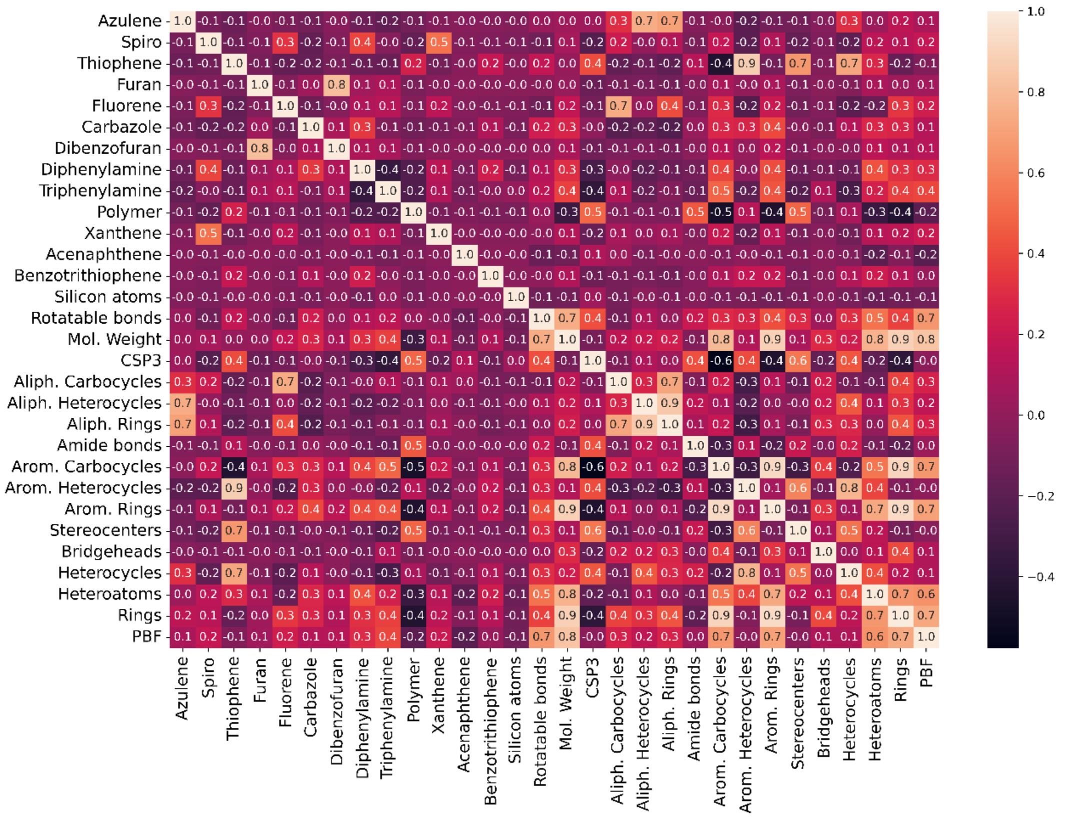

Figure S3. Correlation matrix with the initial 30 structural features in the homogeneous dataset.

We use the same procedure as in the heterogeneous database to perform a recursive feature selection on the remaining 21 structural features of the homogeneous database, as shown in Figure S4, where we drop the following features sequentially: diphenylamine, amide bonds, carbazole, aromatic carbocycles, bridgehead atoms, silicon atoms, xanthene, furan, molecular planarity, polymer, aromatic heterocycles, aliphatic heterocycles, stereocenters, heteroatoms, aliphatic carbocycles, benzotrithiophene, rotatable bonds, spiro atoms, triphenylamine. We can observe how the optimum number of structural features is reached with nine features:

1. Spiro atoms
2. Carbazole
3. Triphenylamine
4. Acenaphthene
5. Benzotrithiophene
6. sp^3 Carbons
7. Aliphatic heterocycles
8. Aromatic heterocycles
9. Molecular planarity (PBF)[1]

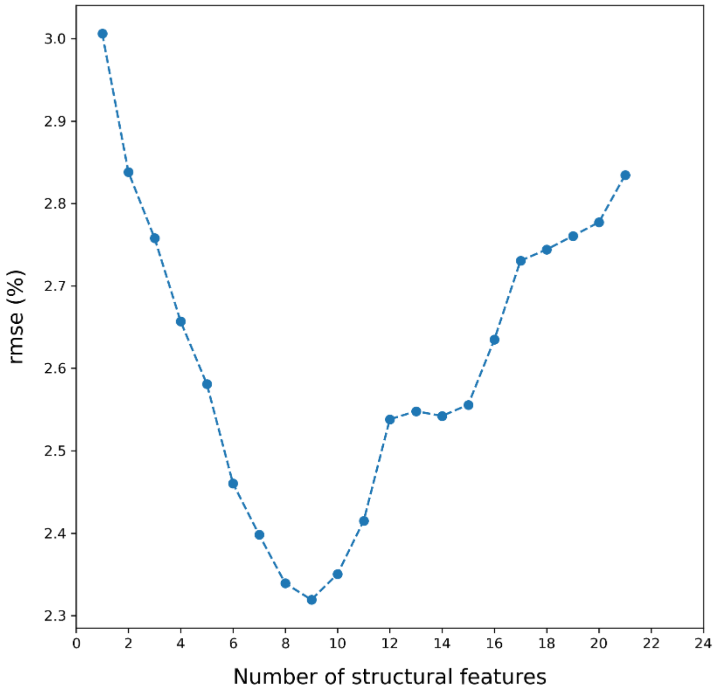

Figure S4. rmse values obtained with a feature recursive elimination in the homogeneous database.

b. Electronic features

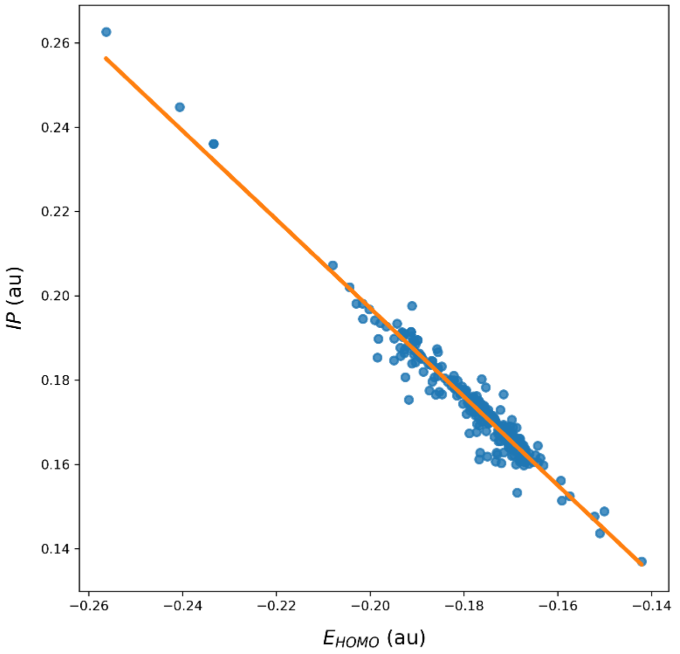

Figure S5. Ionization potential (IP) and HOMO energy data in the heterogeneous database.

S2. Conformer search

Starting from the SMILES string of each HTM, we calculated the ensemble of the ten most stable conformers with a UFF forcefield, using Open Babel.[2] Then, we optimized these ten geometries using PM7[3] with Gaussian16,[4] and the most stable geometry was used as a starting point for the subsequent density functional theory (DFT) calculations.

S3. ML model

a. Differential evolution algorithm

We used a differential evolution algorithm,[5] as implemented in SciPy,[6] using a population size of 15 per parameter, a recombination rate of 0.7 and a mutation of 0.5-1.0.

b. Heterogeneous database parameters

Table S1. Optimized parameters and resulting *rmse* and correlation coefficient (*r*) values with the heterogeneous database (values within parentheses correspond to the test set, and those outside of parentheses correspond to the training set).

γ_{fam}	γ_{arch}	γ_{fp}	γ_{str}	γ_{elec}	γ_{add}	α	rmse	r
0.179072	0.154827	1.0	0.000012	0.000640	0.020764	0.431047	3.0% (3.0%)	0.68 (0.72)

c. Homogeneous database parameters

Table S2. Optimized parameters and resulting *rmse* and *r* values with the homogeneous database (values within parentheses correspond to the test set, and those outside of parentheses correspond to the training set).

Type	γ_{fp}	γ_{str}	γ_{elec}	γ_{add}	α	rmse	r
Fingerprint + Additives	1.0	0	0	0.129438	0.042468	2.8% (2.8%)	0.59 (0.54)
Structural + Additives	0	1.0	0	0.030761	0.004050	6.9% (3.9%)	0.19 (0.27)
Electronic + Additives	0	0	1.0	0.151269	0.022335	4.1% (5.4%)	0.40 (0.65)
Fingerprint + Structural + Electronic + Additives	1.0	0.000155	0.000026	0.071131	0.040924	2.8% (2.7%)	0.59 (0.57)

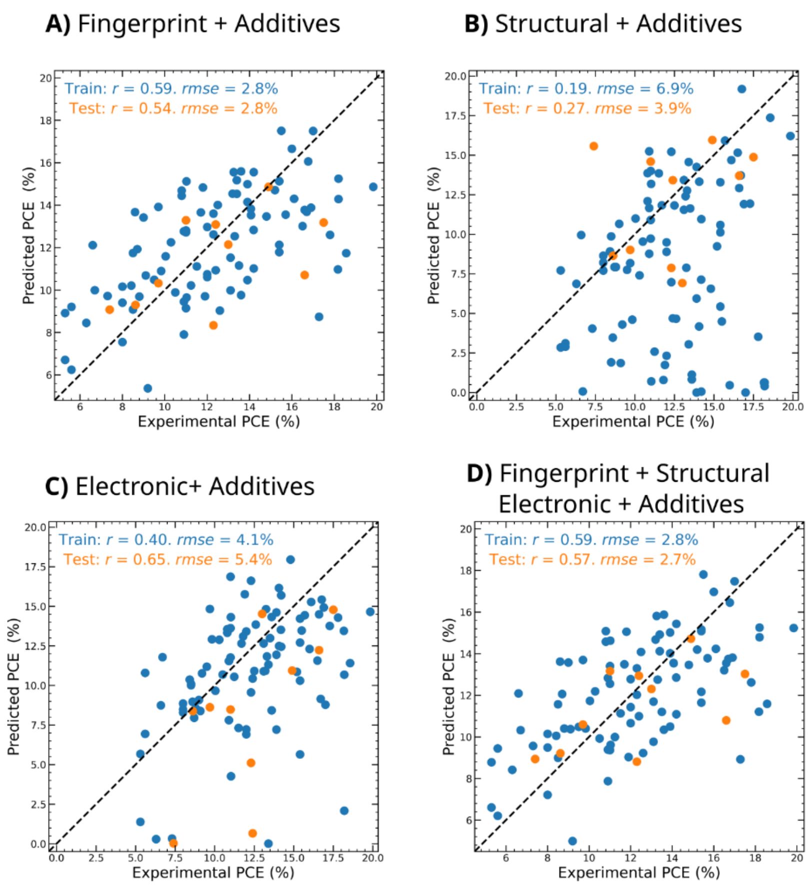

Figure S6. Experimental and predicted PCE of data in the homogeneous database, when using different types of features.

S4. kNN results

Using the *k*-nearest neighbours algorithm, we obtain the following results with the homogeneous dataset, which present a similar trend to the KRR results.

Table S3. Optimized parameters and resulting *rmse* and *r* values with the homogeneous database using kNN (values within parentheses correspond to the test set, and those outside of parentheses correspond to the training set).

Type	γ_{fp}	γ_{str}	γ_{elec}	γ_{add}	k	rmse	r
Fingerprint + Structural + Electronic + Additives	7.637	7.403	0.677	2.664	3	2.4% (2.1%)	0.73 (0.77)

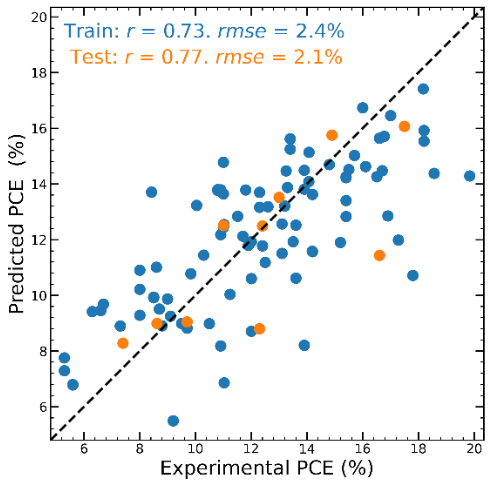

Figure S7. Experimental and predicted PCE of data in the homogeneous database sing kNN, when using different types of features.

S5. Chemical fragments correlation with PCE

Given than most of the model performance is due to the fingerprints, we can analyse which bits of the fingerprints are more correlated with PCE.

For each bit, we created an array of length *N* (where *N* is the number of molecules in the dataset), which will have values of either 0 (bit absent in that molecule) or 1 (bit present in that molecule). Then, we can study the correlation of these with an array containing the PCE values of each molecule in the dataset. We used a point biserial correlation coefficient (*r*), which is equivalent to Pearson's correlation when one variable is binary. We give this value, its corresponding p-value (as the probability of observing the same or larger |*r*| if data is uncorrelated) and the 95% confidence interval below, for the 10 fragments with the largest |*r*| values.

Additionally, we have performed a Mann-Whitney U test for each fragment, which measures the statistical difference between two arrays: $\boldsymbol{PCE}_1$ and $\boldsymbol{PCE}_0$, where $\boldsymbol{PCE}_1$ contains the PCE values of the molecules with that fragment present and $\boldsymbol{PCE}_0$ contains the PCE values of the molecules with that fragment absent. The Mann-Whitney U statistics is defined as:

$$U = \sum_{i=1}^{n}\sum_{j=1}^{m} S(X_i, Y_i)$$

with $S(X,Y) = \begin{cases} 1 & if\ X > Y \\ 1/2 & if\ X = Y \\ 0 & if\ X < Y \end{cases}$. We report in the table below the U_1 and U_2 statistics, where $U_1 = \sum_{i=1}^{n}\sum_{j=1}^{m} S(PCE_{1,i}, PCE_{0,j})$ and $U_2 = \sum_{i=1}^{n}\sum_{j=1}^{m} S(PCE_{0,i}, PCE_{1,j})$. For example, a large value of U_1 indicates that there is a significant difference between the $\boldsymbol{PCE}_0$ and $\boldsymbol{PCE}_1$ arrays, with $\boldsymbol{PCE}_1$ having larger values.

The results for the 19 molecules with $|r| > 0.3$ in the homogeneous database are shown in Table S3, and the results for the 27 molecules with $|r| > 0.2$ in the heterogeneous database are shown in Table S4.

Table S4. Results for the bits whose correlation coefficient $|r| > 0.3$ with respect to PCE for all molecules in the homogeneous dataset, with its corresponding p-value and 95% confidence interval, as well as the Mann-Whitney U test values (U_1 and U_2) and its associated p-value for each fragment.

Homogeneous database						
**Bit**	***r***	**p-value**	***r* 95% interval**	**U_1**	**U_2**	**p-value**
**832** 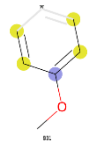	0.47	1.08E-06	[0.30,0.61]	1919.5	576.5	3.65E-06
**842** 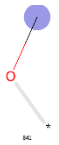	0.42	1.25E-05	[0.25,0.57]	1837.5	637.5	3.26E-05
**1537** 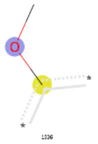	0.42	1.25E-05	[0.25,0.57]	1837.5	637.5	3.26E-05
**719** 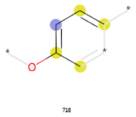	0.41	2.53E-05	[0.23,0.56]	1807.5	656.5	6.51E-05
**15** 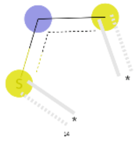	-0.41	2.85E-05	[-0.56,-0.23]	162	817	3.15E-04
**323** 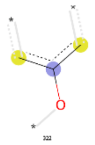	0.40	3.03E-05	[0.23,0.56]	1792.5	658.5	7.99E-05
**171** 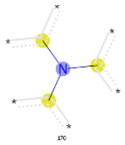	0.40	4.31E-05	[0.22,0.55]	1670.5	604.5	1.19E-04
**882** 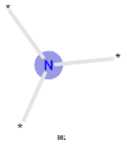	0.40	4.31E-05	[0.22,0.55]	1670.5	604.5	1.19E-04

**961** 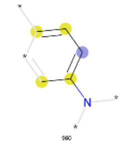	0.40	4.31E-05	[0.22,0.55]	1670.5	604.5	1.19E-04
**1097** 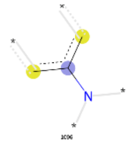	0.40	4.31E-05	[0.22,0.55]	1670.5	604.5	1.19E-04
**1568** 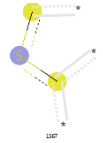	-0.38	8.35E-05	[-0.54,-0.20]	207.5	848.5	0.000687
**782** 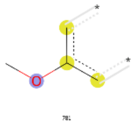	0.37	1.32E-04	[0.19,0.53]	1751.5	699.5	0.000253
**2033** 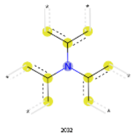	0.35	3.83E-04	[0.16,0.51]	1686.5	732.5	0.000838
**740** 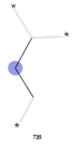	-0.34	4.95E-04	[-0.50,-0.16]	336	1008	0.001605
**2** 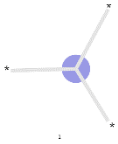	-0.33	7.90E-04	[-0.49,-0.14]	438	1162	0.001836
**1212** 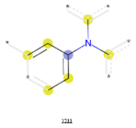	0.33	7.95E-04	[0.14,0.49]	1271.5	444.5	0.000588
**123** 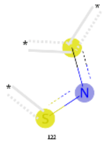	-0.33	8.55E-04	[-0.49,-0.14]	135.5	515.5	0.010454
**1445** 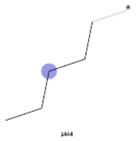	-0.31	1.51E-03	[-0.48,-0.12]	693	1582	0.001331

**1936** 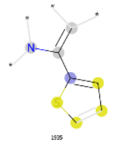	-0.30	2.28E-03	[-0.47,-0.11]	88	476	0.004971

Table S5. Results for the bits whose correlation coefficient $|r| > 0.2$ with respect to PCE for all molecules in the heterogeneous dataset, with its corresponding p-value and 95% confidence interval, as well as the Mann-Whitney U test values (U_1 and U_2) and its associated p-value for each fragment.

Heterogeneous database						
**Bit**	***r***	**p-value**	***r* 95% interval**	**U_1**	**U_2**	**p-value**
**1097** 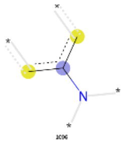	0.32	1.24E-07	[0.20,0.42]	10077	4821	5.67E-06
**882** 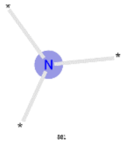	0.30	6.73E-07	[0.18,0.40]	9993.5	5126.5	3.03E-05
**842** 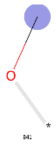	0.27	8.14E-06	[0.15,0.38]	11279	6211	5.37E-05
**1936** 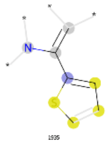	-0.27	9.58E-06	[-0.37,-0.15]	125	1453	4.29E-04
**171** 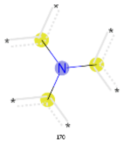	0.27	1.03E-05	[0.15,0.37]	9875	5665	3.72 E-04
**961** 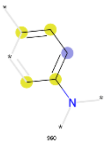	0.26	1.32E-05	[0.15,0.37]	9906	5634	3.04 E-04
**1537** 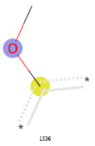	0.26	1.94E-05	[0.14,0.37]	11173.5	6364.5	1.30 E-04
**832** 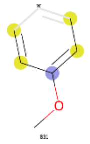	0.25	2.48E-05	[0.14,0.36]	11256.5	6677.5	3.14 E-04

**2033**<br>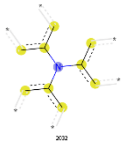	0.24	5.21E-05	[0.13,0.35]	10357	6251	7.86 E-04
**323**<br>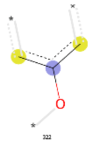	0.24	8.19E-05	[0.12,0.35]	10665	6495	7.94 E-04
**1446**<br>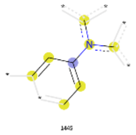	-0.24	8.47E-05	[-0.35,0.12]	609	2475	4.00E-04
**719**<br>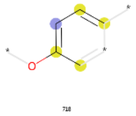	0.23	1.24E-04	[0.12,0.34]	10691.5	6642.5	1.19E-03
**2**<br>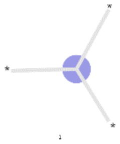	-0.22	2.45E-04	[-0.33,-0.11]	3266	6082	2.15E-03
**740**<br>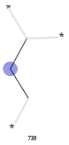	-0.22	2.62E-04	[-0.33,0.11]	2744.5	5245.5	3.20E-03
**335**<br>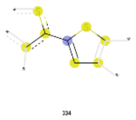	-0.22	2.92E-04	[-0.33,-0.10]	178	1400	1.20E-03
**9**<br>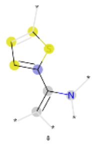	-0.22	2.99E-04	[-0.33,-0.10]	94	966	4.80E-03
**1092**<br>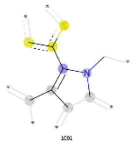	-0.22	2.99E-04	[-0.33,-0.10]	94	966	4.80E-03
**1094**<br>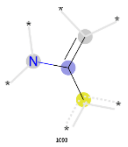	-0.22	2.99E-04	[-0.33,-0.10]	94	966	4.80E-03

**1273** 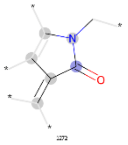	-0.22	2.99E-04	[-0.33,-0.10]	94	966	4.80E-03
**1815** 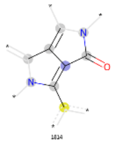	-0.22	2.99E-04	[-0.33,-0.10]	94	966	4.80E-03
**364** 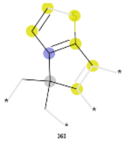	-0.22	3.82E-04	[-0.33,-0.10]	4	530	1.66E-02
**1552** 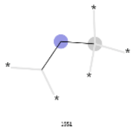	-0.22	3.82E-04	[-0.33,-0.10]	4	530	1.66E-02
**2008** 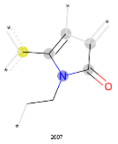	-0.21	5.71E-04	[-0.32,-0.09]	8	526	1.84E-02
**1172** 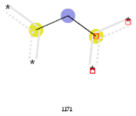	-0.21	6.80E-04	[-0.32,-0.09]	94.5	965.5	4.85E-03
**592** 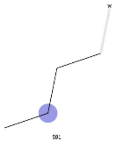	-0.20	9.07E-04	[-0.31,-0.08]	6542	10288	2.34E-03
**1912** 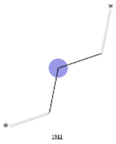	-0.20	9.07E-04	[-0.31,-0.08]	6542	10288	2.34E-03
**758** 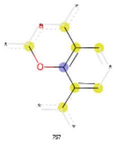	-0.20	9.60E-04	[-0.31,-0.09]	11.5	522.5	2.00E-02

S6. Database

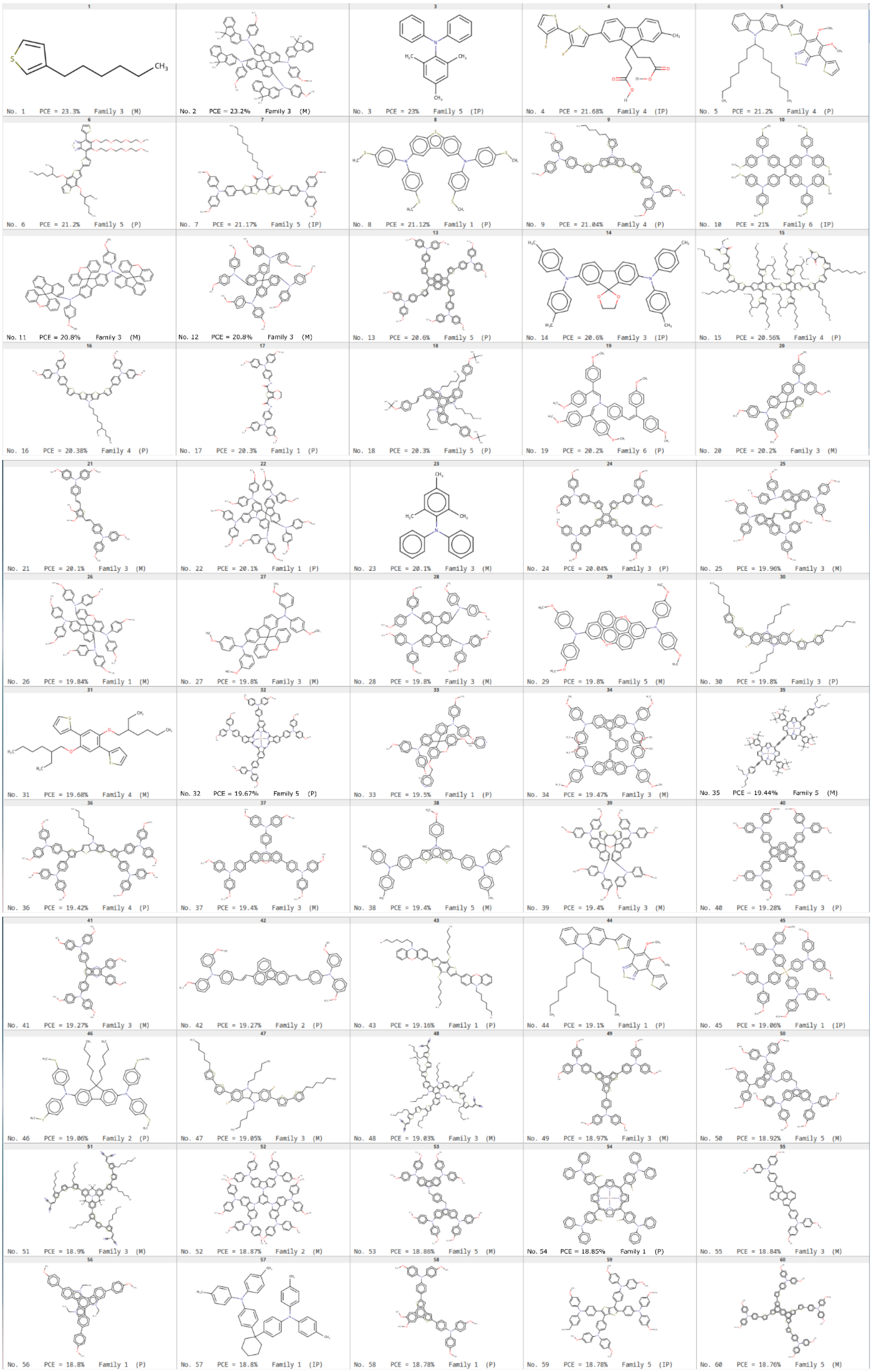
No. 1 PCE = 23.3% Family 3 (M)
No. 2 PCE = 23.2% Family 3 (M)
No. 3 PCE = 23% Family 5 (IP)
No. 4 PCE = 21.68% Family 4 (IP)
No. 5 PCE = 21.2% Family 4 (P)
No. 6 PCE = 21.2% Family 5 (P)
No. 7 PCE = 21.17% Family 5 (IP)
No. 8 PCE = 21.12% Family 1 (P)
No. 9 PCE = 21.04% Family 4 (P)
No. 10 PCE = 21% Family 6 (IP)
No. 11 PCE = 20.8% Family 3 (M)
No. 12 PCE = 20.8% Family 3 (M)
No. 13 PCE = 20.6% Family 5 (P)
No. 14 PCE = 20.6% Family 3 (IP)
No. 15 PCE = 20.56% Family 4 (P)
No. 16 PCE = 20.38% Family 4 (P)
No. 17 PCE = 20.3% Family 1 (P)
No. 18 PCE = 20.3% Family 5 (P)
No. 19 PCE = 20.2% Family 6 (P)
No. 20 PCE = 20.2% Family 3 (M)
No. 21 PCE = 20.1% Family 3 (M)
No. 22 PCE = 20.1% Family 1 (P)
No. 23 PCE = 20.1% Family 3 (M)
No. 24 PCE = 20.04% Family 3 (P)
No. 25 PCE = 19.96% Family 3 (M)
No. 26 PCE = 19.84% Family 1 (M)
No. 27 PCE = 19.8% Family 3 (M)
No. 28 PCE = 19.8% Family 3 (M)
No. 29 PCE = 19.8% Family 5 (M)
No. 30 PCE = 19.8% Family 3 (P)
No. 31 PCE = 19.68% Family 4 (M)
No. 32 PCE = 19.67% Family 5 (P)
No. 33 PCE = 19.5% Family 1 (P)
No. 34 PCE = 19.47% Family 3 (M)
No. 35 PCE = 19.44% Family 5 (M)
No. 36 PCE = 19.42% Family 4 (P)
No. 37 PCE = 19.4% Family 3 (M)
No. 38 PCE = 19.4% Family 5 (M)
No. 39 PCE = 19.4% Family 3 (M)
No. 40 PCE = 19.28% Family 3 (P)
No. 41 PCE = 19.27% Family 3 (M)
No. 42 PCE = 19.27% Family 2 (P)
No. 43 PCE = 19.16% Family 1 (P)
No. 44 PCE = 19.1% Family 1 (P)
No. 45 PCE = 19.06% Family 1 (IP)
No. 46 PCE = 19.06% Family 2 (P)
No. 47 PCE = 19.05% Family 3 (M)
No. 48 PCE = 19.03% Family 3 (M)
No. 49 PCE = 18.97% Family 3 (M)
No. 50 PCE = 18.92% Family 5 (M)
No. 51 PCE = 18.9% Family 3 (M)
No. 52 PCE = 18.87% Family 2 (M)
No. 53 PCE = 18.86% Family 5 (M)
No. 54 PCE = 18.85% Family 1 (P)
No. 55 PCE = 18.84% Family 3 (M)
No. 56 PCE = 18.8% Family 1 (P)
No. 57 PCE = 18.8% Family 1 (IP)
No. 58 PCE = 18.78% Family 1 (P)
No. 59 PCE = 18.78% Family 5 (IP)
No. 60 PCE = 18.76% Family 5 (M)

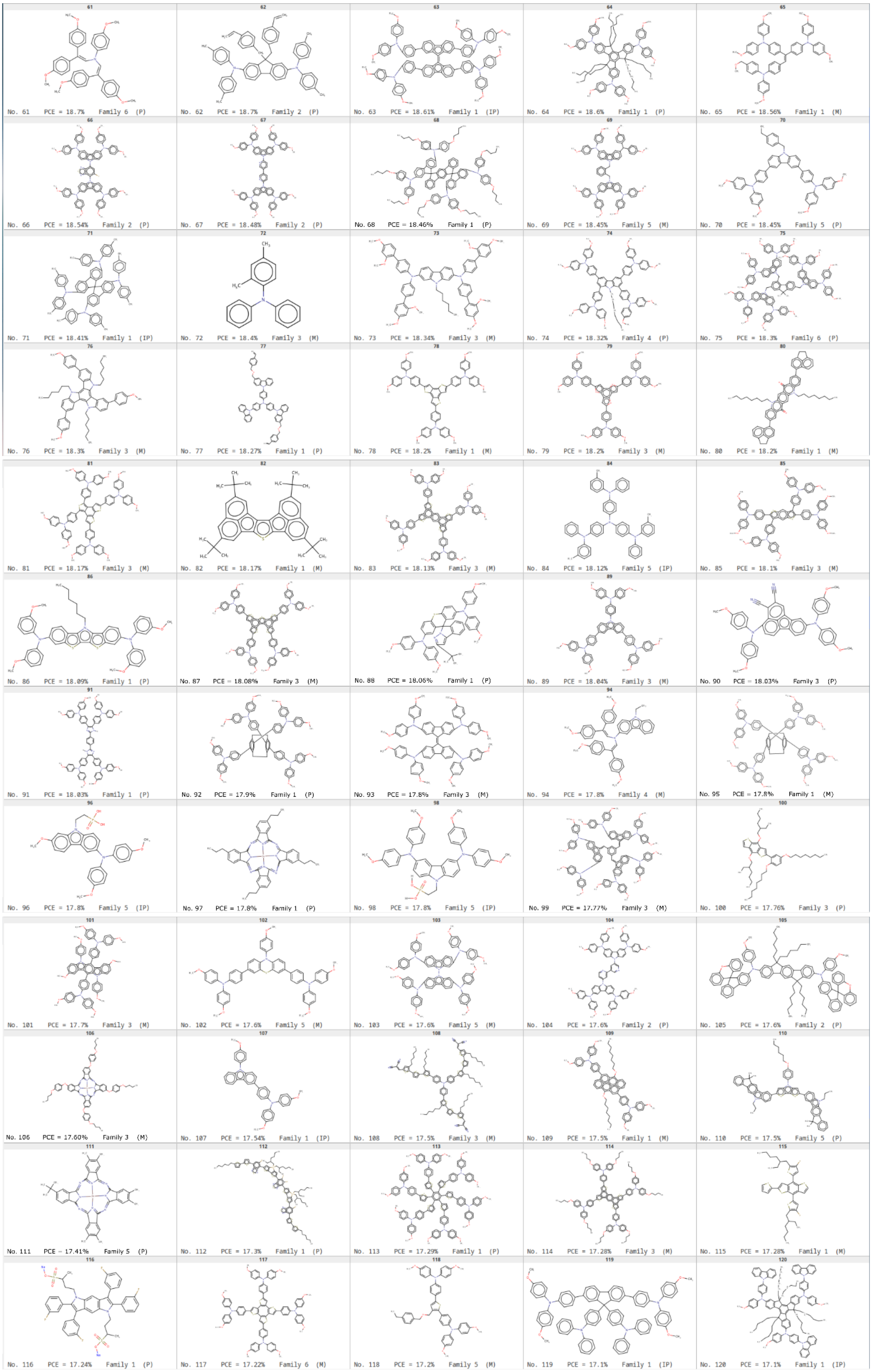
No. 61 PCE = 18.7% Family 6 (P)
No. 62 PCE = 18.7% Family 2 (P)
No. 63 PCE = 18.61% Family 1 (IP)
No. 64 PCE = 18.6% Family 1 (P)
No. 65 PCE = 18.56% Family 1 (M)
No. 66 PCE = 18.54% Family 2 (P)
No. 67 PCE = 18.48% Family 2 (P)
No. 68 PCE = 18.46% Family 1 (P)
No. 69 PCE = 18.45% Family 5 (M)
No. 70 PCE = 18.45% Family 5 (P)
No. 71 PCE = 18.41% Family 1 (IP)
No. 72 PCE = 18.4% Family 3 (M)
No. 73 PCE = 18.34% Family 3 (M)
No. 74 PCE = 18.32% Family 4 (P)
No. 75 PCE = 18.3% Family 6 (P)
No. 76 PCE = 18.3% Family 3 (M)
No. 77 PCE = 18.27% Family 1 (P)
No. 78 PCE = 18.2% Family 1 (M)
No. 79 PCE = 18.2% Family 3 (M)
No. 80 PCE = 18.2% Family 1 (M)
No. 81 PCE = 18.17% Family 3 (M)
No. 82 PCE = 18.17% Family 1 (M)
No. 83 PCE = 18.13% Family 3 (M)
No. 84 PCE = 18.12% Family 5 (IP)
No. 85 PCE = 18.1% Family 3 (M)
No. 86 PCE = 18.09% Family 1 (P)
No. 87 PCE = 18.08% Family 3 (M)
No. 88 PCE = 18.06% Family 1 (P)
No. 89 PCE = 18.04% Family 3 (M)
No. 90 PCE = 18.03% Family 3 (P)
No. 91 PCE = 18.03% Family 1 (P)
No. 92 PCE = 17.9% Family 1 (P)
No. 93 PCE = 17.8% Family 3 (M)
No. 94 PCE = 17.8% Family 4 (M)
No. 95 PCE = 17.8% Family 1 (M)
No. 96 PCE = 17.8% Family 5 (IP)
No. 97 PCE = 17.8% Family 1 (P)
No. 98 PCE = 17.8% Family 5 (IP)
No. 99 PCE = 17.77% Family 3 (M)
No. 100 PCE = 17.76% Family 3 (P)
No. 101 PCE = 17.7% Family 3 (M)
No. 102 PCE = 17.6% Family 5 (M)
No. 103 PCE = 17.6% Family 5 (M)
No. 104 PCE = 17.6% Family 2 (P)
No. 105 PCE = 17.6% Family 2 (P)
No. 106 PCE = 17.60% Family 3 (M)
No. 107 PCE = 17.54% Family 1 (IP)
No. 108 PCE = 17.5% Family 3 (M)
No. 109 PCE = 17.5% Family 1 (M)
No. 110 PCE = 17.5% Family 5 (P)
No. 111 PCE = 17.41% Family 5 (P)
No. 112 PCE = 17.3% Family 1 (P)
No. 113 PCE = 17.29% Family 1 (P)
No. 114 PCE = 17.28% Family 3 (M)
No. 115 PCE = 17.28% Family 1 (M)
No. 116 PCE = 17.24% Family 1 (P)
No. 117 PCE = 17.22% Family 6 (M)
No. 118 PCE = 17.2% Family 5 (M)
No. 119 PCE = 17.1% Family 1 (IP)
No. 120 PCE = 17.1% Family 1 (IP)

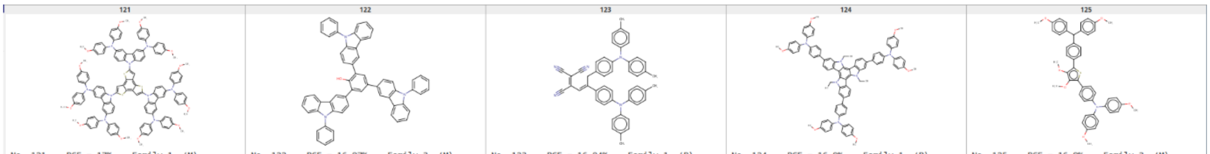

No. 121 PCE = 17% Family 1 (M)
No. 122 PCE = 16.97% Family 3 (M)
No. 123 PCE = 16.94% Family 1 (P)
No. 124 PCE = 16.9% Family 1 (P)
No. 125 PCE = 16.9% Family 3 (M)

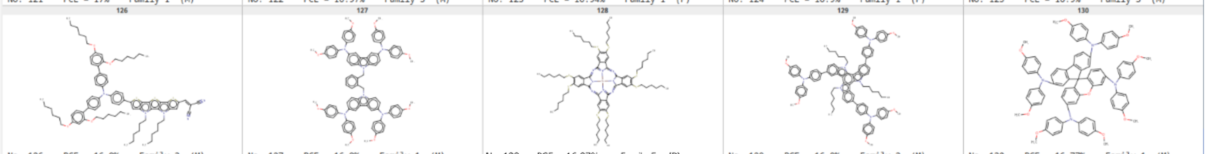

No. 126 PCE = 16.9% Family 3 (M)
No. 127 PCE = 16.9% Family 1 (M)
No. 128 PCE = 16.87% Family 5 (P)
No. 129 PCE = 16.8% Family 3 (M)
No. 130 PCE = 16.77% Family 1 (M)

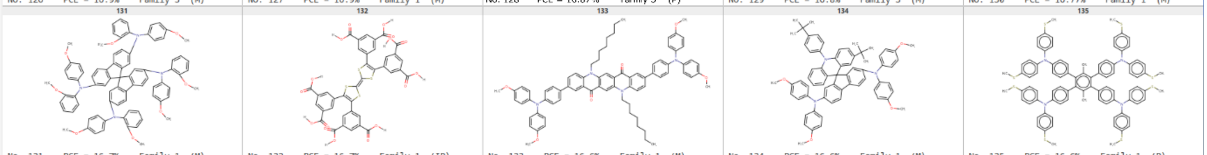

No. 131 PCE = 16.7% Family 1 (M)
No. 132 PCE = 16.7% Family 1 (IP)
No. 133 PCE = 16.6% Family 1 (M)
No. 134 PCE = 16.6% Family 1 (M)
No. 135 PCE = 16.6% Family 1 (P)

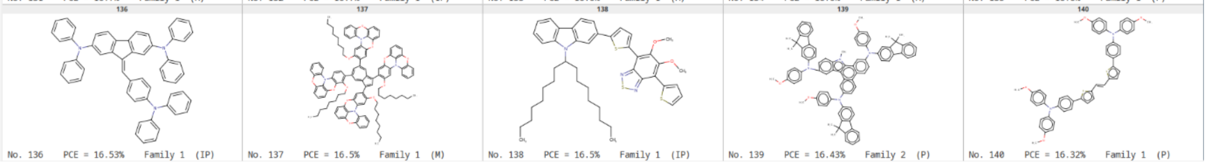

No. 136 PCE = 16.53% Family 1 (IP)
No. 137 PCE = 16.5% Family 1 (M)
No. 138 PCE = 16.5% Family 1 (IP)
No. 139 PCE = 16.43% Family 2 (P)
No. 140 PCE = 16.32% Family 1 (P)

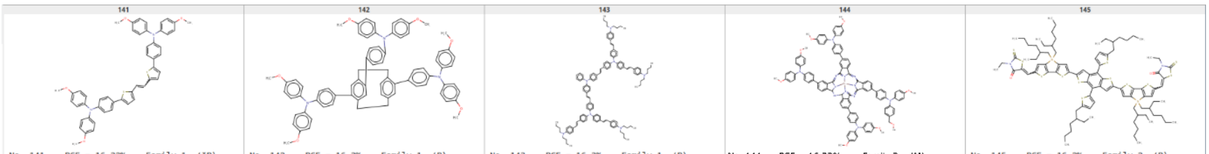

No. 141 PCE = 16.32% Family 1 (IP)
No. 142 PCE = 16.3% Family 1 (P)
No. 143 PCE = 16.3% Family 1 (P)
No. 144 PCE = 16.23% Family 3 (M)
No. 145 PCE = 16.2% Family 2 (P)

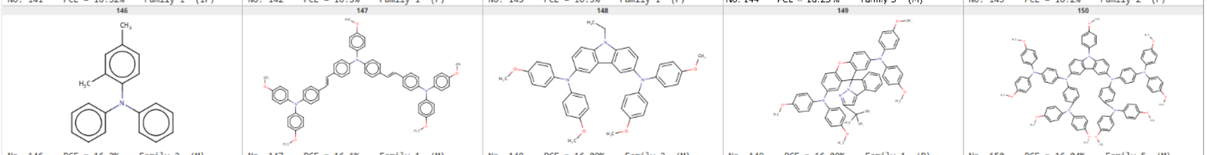

No. 146 PCE = 16.2% Family 2 (M)
No. 147 PCE = 16.1% Family 1 (M)
No. 148 PCE = 16.09% Family 3 (M)
No. 149 PCE = 16.08% Family 1 (P)
No. 150 PCE = 16.04% Family 5 (M)

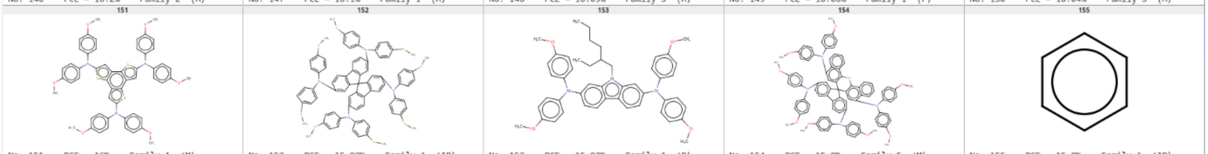

No. 151 PCE = 16% Family 1 (M)
No. 152 PCE = 15.92% Family 1 (IP)
No. 153 PCE = 15.92% Family 1 (P)
No. 154 PCE = 15.9% Family 5 (M)
No. 155 PCE = 15.8% Family 1 (IP)

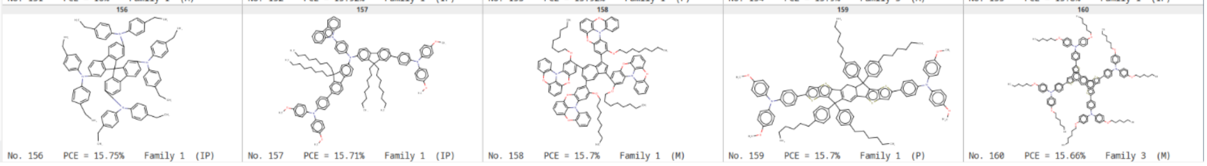

No. 156 PCE = 15.75% Family 1 (IP)
No. 157 PCE = 15.71% Family 1 (IP)
No. 158 PCE = 15.7% Family 1 (M)
No. 159 PCE = 15.7% Family 1 (P)
No. 160 PCE = 15.66% Family 3 (M)

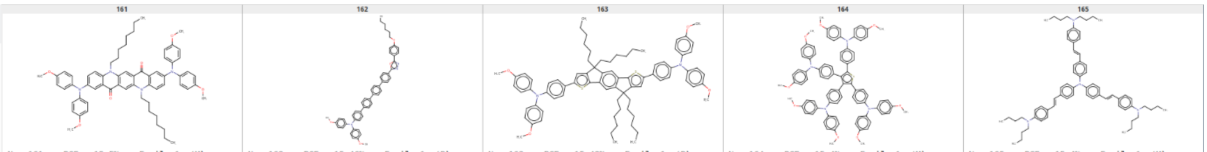

No. 161 PCE = 15.5% Family 1 (M)
No. 162 PCE = 15.46% Family 1 (P)
No. 163 PCE = 15.43% Family 1 (P)
No. 164 PCE = 15.4% Family 1 (M)
No. 165 PCE = 15.4% Family 1 (M)

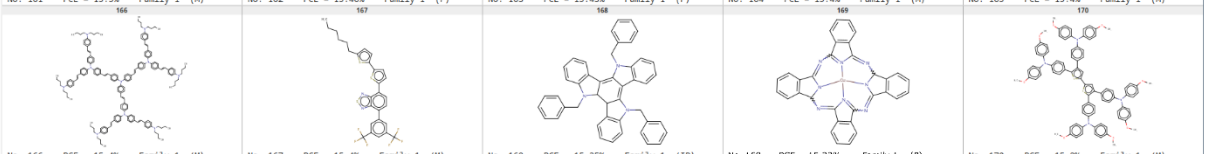

No. 166 PCE = 15.4% Family 1 (M)
No. 167 PCE = 15.4% Family 1 (M)
No. 168 PCE = 15.35% Family 1 (IP)
No. 169 PCE = 15.33% Family 1 (P)
No. 170 PCE = 15.2% Family 1 (M)

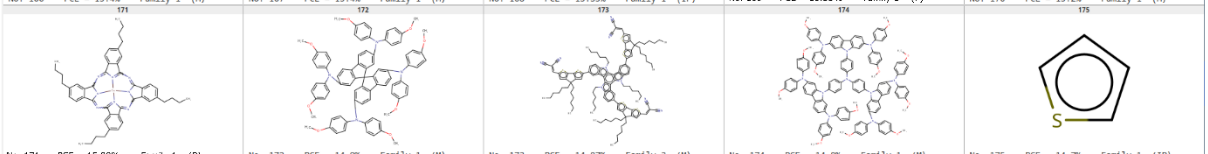

No. 171 PCE = 15.00% Family 1 (P)
No. 172 PCE = 14.9% Family 1 (M)
No. 173 PCE = 14.87% Family 3 (M)
No. 174 PCE = 14.8% Family 1 (M)
No. 175 PCE = 14.7% Family 1 (IP)

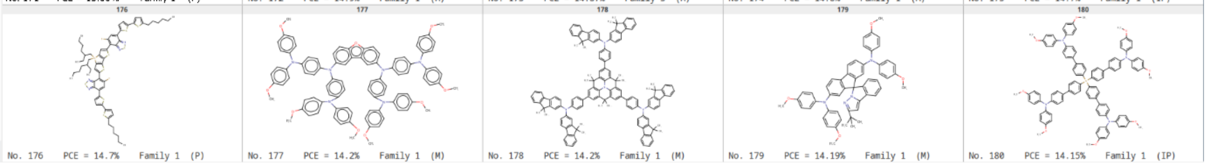

No. 176 PCE = 14.7% Family 1 (P)
No. 177 PCE = 14.2% Family 1 (M)
No. 178 PCE = 14.2% Family 1 (M)
No. 179 PCE = 14.19% Family 1 (M)
No. 180 PCE = 14.15% Family 1 (IP)

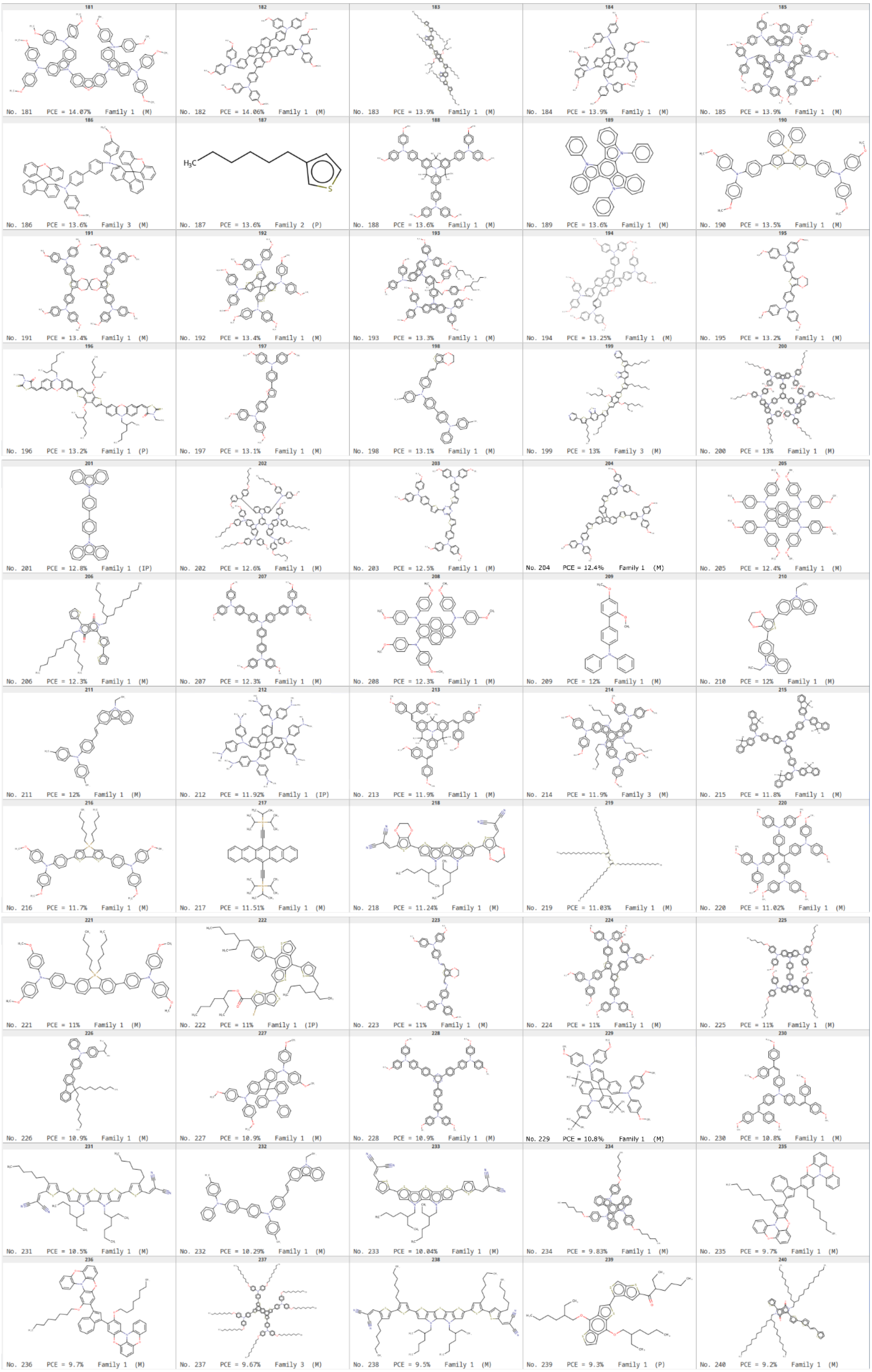
No. 181 PCE = 14.07% Family 1 (M)
No. 182 PCE = 14.06% Family 1 (M)
No. 183 PCE = 13.9% Family 1 (M)
No. 184 PCE = 13.9% Family 1 (M)
No. 185 PCE = 13.9% Family 1 (M)
No. 186 PCE = 13.6% Family 3 (M)
No. 187 PCE = 13.6% Family 2 (P)
No. 188 PCE = 13.6% Family 1 (M)
No. 189 PCE = 13.6% Family 1 (M)
No. 190 PCE = 13.5% Family 1 (M)
No. 191 PCE = 13.4% Family 1 (M)
No. 192 PCE = 13.4% Family 1 (M)
No. 193 PCE = 13.3% Family 1 (M)
No. 194 PCE = 13.25% Family 1 (M)
No. 195 PCE = 13.2% Family 1 (M)
No. 196 PCE = 13.2% Family 1 (P)
No. 197 PCE = 13.1% Family 1 (M)
No. 198 PCE = 13.1% Family 1 (M)
No. 199 PCE = 13% Family 3 (M)
No. 200 PCE = 13% Family 1 (M)
No. 201 PCE = 12.8% Family 1 (IP)
No. 202 PCE = 12.6% Family 1 (M)
No. 203 PCE = 12.5% Family 1 (M)
No. 204 PCE = 12.4% Family 1 (M)
No. 205 PCE = 12.4% Family 1 (M)
No. 206 PCE = 12.3% Family 1 (M)
No. 207 PCE = 12.3% Family 1 (M)
No. 208 PCE = 12.3% Family 1 (M)
No. 209 PCE = 12% Family 1 (M)
No. 210 PCE = 12% Family 1 (M)
No. 211 PCE = 12% Family 1 (M)
No. 212 PCE = 11.92% Family 1 (IP)
No. 213 PCE = 11.9% Family 1 (M)
No. 214 PCE = 11.9% Family 3 (M)
No. 215 PCE = 11.8% Family 1 (M)
No. 216 PCE = 11.7% Family 1 (M)
No. 217 PCE = 11.51% Family 1 (M)
No. 218 PCE = 11.24% Family 1 (M)
No. 219 PCE = 11.03% Family 1 (M)
No. 220 PCE = 11.02% Family 1 (M)
No. 221 PCE = 11% Family 1 (M)
No. 222 PCE = 11% Family 1 (IP)
No. 223 PCE = 11% Family 1 (M)
No. 224 PCE = 11% Family 1 (M)
No. 225 PCE = 11% Family 1 (M)
No. 226 PCE = 10.9% Family 1 (M)
No. 227 PCE = 10.9% Family 1 (M)
No. 228 PCE = 10.9% Family 1 (M)
No. 229 PCE = 10.8% Family 1 (M)
No. 230 PCE = 10.8% Family 1 (M)
No. 231 PCE = 10.5% Family 1 (M)
No. 232 PCE = 10.29% Family 1 (M)
No. 233 PCE = 10.04% Family 1 (M)
No. 234 PCE = 9.83% Family 1 (M)
No. 235 PCE = 9.7% Family 1 (M)
No. 236 PCE = 9.7% Family 1 (M)
No. 237 PCE = 9.67% Family 3 (M)
No. 238 PCE = 9.5% Family 1 (M)
No. 239 PCE = 9.3% Family 1 (P)
No. 240 PCE = 9.2% Family 1 (M)

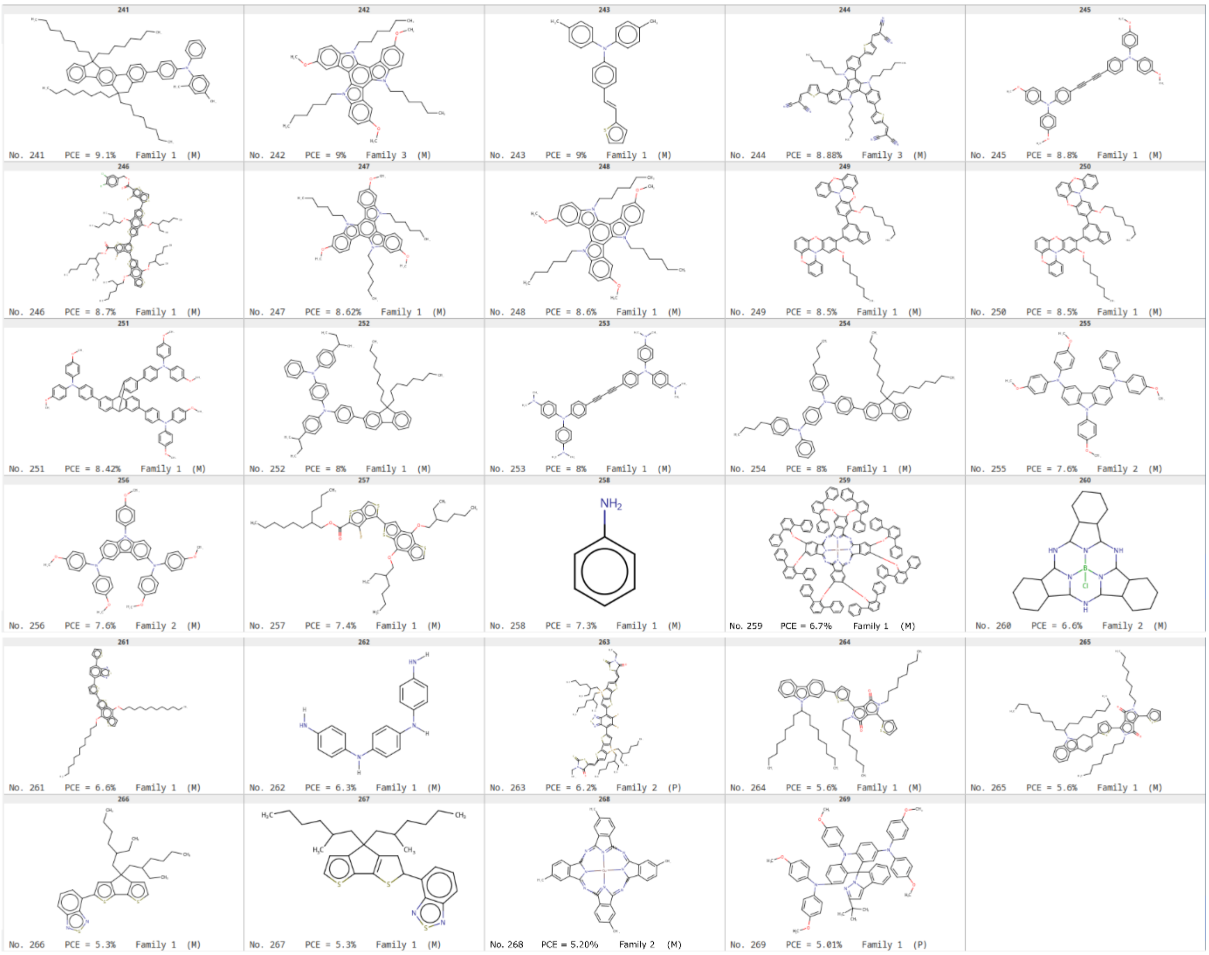

Figure S8. Structure of all HTMs in our database. Labels include the PCE and perovskite family/architecture of each device. In case of polymeric HTMs, we show the structure of the repeating unit.

S7. References

(1) Firth, N. C.; Brown, N.; Blagg, J. Plane of Best Fit: A Novel Method to Characterize the Three-Dimensionality of Molecules. *J. Chem. Inf. Model.* **2012**, *52*, 2516–2525.

(2) O'Boyle, N. M.; Banck, M.; James, C. A.; Morley, C.; Vandermeersch, T.; Hutchison, G. R. Open Babel: An Open Chemical Toolbox. *J. Cheminformatics* **2011**, *3*, 33.

(3) Stewart, J. J. P. Optimization of Parameters for Semiempirical Methods VI: More Modifications to the NDDO Approximations and Re-Optimization of Parameters. *J. Mol. Model.* **2013**, *19*, 1–32.

(4) Frisch, M. J.; Trucks, G. W.; Schlegel, H. B.; Scuseria, G. E.; Robb, M. A.; Cheeseman, J. R.; Scalmani, G.; Barone, V.; Petersson, G. A.; Nakatsuji, H., et al. *Gaussian 16, Revision C.01*; Gaussian, Inc., Wallingford CT, 2016.

(5) Storn, R.; Price, K. Differential Evolution – A Simple and Efficient Heuristic for Global Optimization over Continuous Spaces. *J. Glob. Optim.* **1997**, *11*, 341–359.

(6) Virtanen, P.; Gommers, R.; Oliphant, T. E.; Haberland, M.; Reddy, T.; Cournapeau, D.; Burovski, E.; Peterson, P.; Weckesser, W.; Bright, J., et al. SciPy 1.0: Fundamental Algorithms for Scientific Computing in Python. *Nat. Methods* **2020**, *17*, 261–272.
